# Cathepsins in Bacteria-Macrophage Interaction: Defenders or Victims of Circumstance?

**DOI:** 10.3389/fcimb.2020.601072

**Published:** 2020-12-04

**Authors:** Lidia Szulc-Dąbrowska, Magdalena Bossowska-Nowicka, Justyna Struzik, Felix N. Toka

**Affiliations:** ^1^ Division of Immunology, Department of Preclinical Sciences, Institute of Veterinary Medicine, Warsaw University of Life Sciences-Szkoła Główna Gospodarstwa Wejskiego, Warsaw, Poland; ^2^ Center for Integrative Mammalian Research, Ross University School of Veterinary Medicine, Basseterre, Saint Kitts and Nevis

**Keywords:** cathepsins, bacteria, macrophages, immune invasion, modulation of immune function

## Abstract

Macrophages are the first encounters of invading bacteria and are responsible for engulfing and digesting pathogens through phagocytosis leading to initiation of the innate inflammatory response. Intracellular digestion occurs through a close relationship between phagocytic/endocytic and lysosomal pathways, in which proteolytic enzymes, such as cathepsins, are involved. The presence of cathepsins in the endo-lysosomal compartment permits direct interaction with and killing of bacteria, and may contribute to processing of bacterial antigens for presentation, an event necessary for the induction of antibacterial adaptive immune response. Therefore, it is not surprising that bacteria can control the expression and proteolytic activity of cathepsins, including their inhibitors – cystatins, to favor their own intracellular survival in macrophages. In this review, we summarize recent developments in defining the role of cathepsins in bacteria-macrophage interaction and describe important strategies engaged by bacteria to manipulate cathepsin expression and activity in macrophages. Particularly, we focus on specific bacterial species due to their clinical relevance to humans and animal health, i.e., *Mycobacterium, Mycoplasma*, *Staphylococcus*, *Streptococcus, Salmonella*, *Shigella, Francisella, Chlamydia, Listeria, Brucella, Helicobacter*, *Neisseria*, and other genera.

## Introduction

Macrophages and neutrophils are major types of phagocytic cells of the innate immune system. They are specialized to detect, engulf and destroy some bacteria and viruses or other foreign particles that can be dangerous to the health and proper functioning of the organism. In the detection and recognition process, a large repertoire of pattern recognition receptors (PRRs) and other molecules expressed on/in macrophages are engaged, ensuring the distinction between “self” and “non-self”, which is fundamental to maintenance of tolerance with simultaneous potential for response to threat by the “non-self” (Mukhopadhyay et al., 2009). Regarding the recognition of bacteria, the target cell is engulfed through extension of pseudopodia around the target and formation of a phagosome, which subsequently fuses with the lysosome to form a phagolysosome. In this compartment, the bacterial cell is exposed to a toxic environment characterized by acidification, presence of active proteolytic and lipolytic enzymes and products of the respiratory burst, including reactive oxygen species (ROS) and reactive nitrogen species (RNS) (Russell et al., 2009; Slauch, 2011; Hirayama et al., 2018). These events eventually lead to destruction of bacteria and allow processing of peptide antigens that are subsequently presented on the major histocompatibility class II (MHC II) molecules leading to activation of helper T (Th) cells, thus stimulating acquired immune response. Therefore, macrophages, as antigen presenting cells (APCs), constitute a bridge between non-specific (innate) and specific (adaptive) immunity (Hirayama et al., 2018).

**Figure 1:**
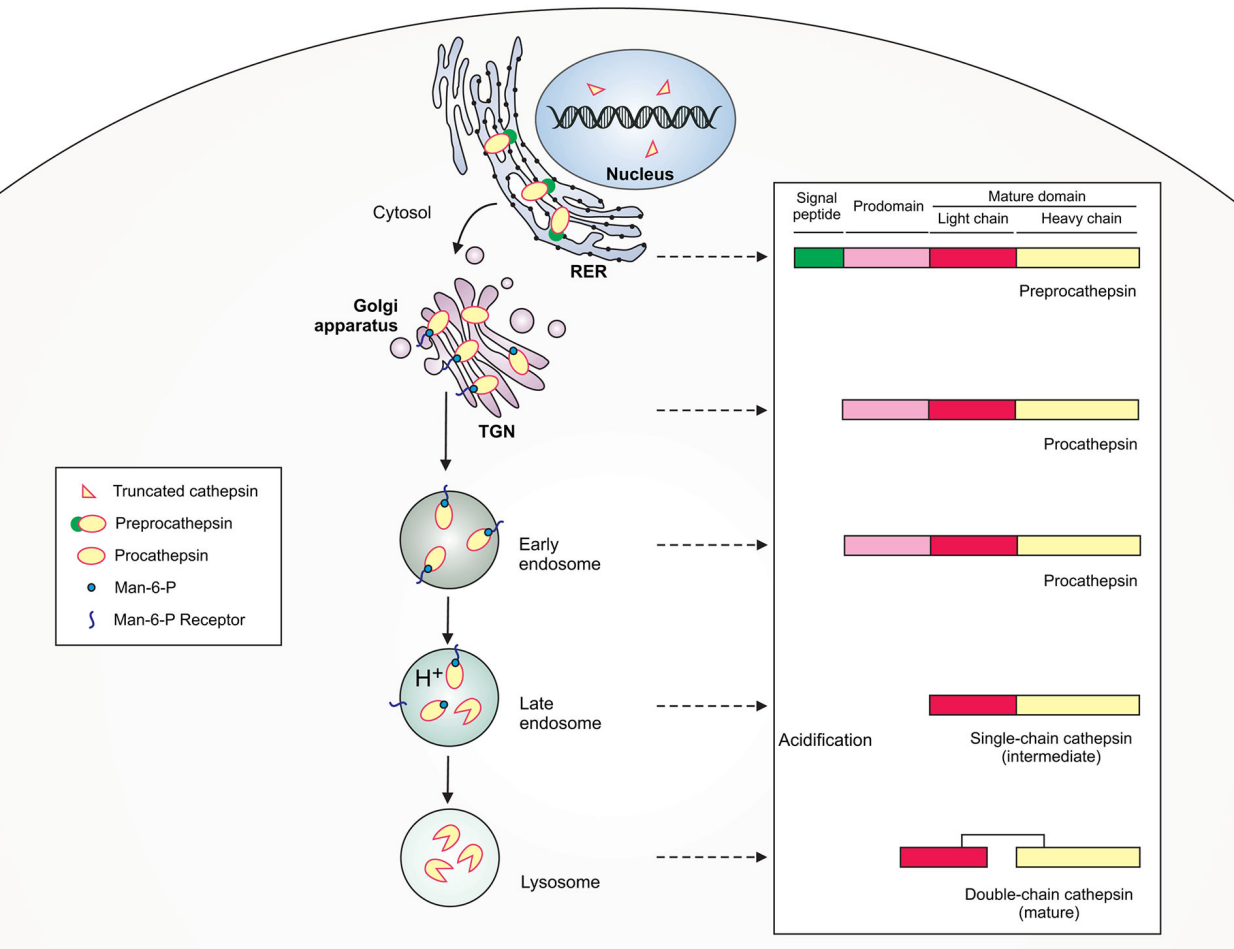
Schematic illustration of lysosomal cathepsin maturation. Cathepsins are synthesized as preproenzymes with the N-terminal signal peptide that targets the protein to the lumen of the rough endoplasmic reticulum (RER). After the removal of the signal peptide (pre) in the ER lumen, the protopeptides use a mannose-6-phosphate (Man-6-P) receptor pathway for delivery *via* the trans-Golgi network (TGN) to early/late endosomes. Within late endosomes the pH drops and procathepsins dissociate from Man-6-P receptor releasing proteolytically active single-chain intermediate cathepsins. Within the lysosome, the single chain protein is further processed *via* autocatalysis into mature two-chain form composed of an N-terminal light chain and a C-terminal heavy chain.

The function of macrophages as effective sentinels, phagocytes and APCs is possible due to the presence of cathepsins (Cts) – serine, aspartic and cysteine peptidases that are involved in regulation of innate (PRRs signaling, pathogen killing, apoptosis) and adaptive (antigen processing and presentation) immune responses (Conus and Simon, 2010). Cathepsins are structurally a heterogeneous group of proteases that were first described as intracellular enzymes of protein degrading activity in a slightly acidic environment (Willstätter and Bamann, 1929). Currently, the name “cathepsin” refers to the two serine proteases (Cts A and G), two aspartic proteases (Cts D and E) and eleven lysosomal cysteine proteases (Cts B, C, F, H, K, L, O, S, V, X, and W) (Turk et al., 2001; Rossi et al., 2004). Cysteine proteases have a similar structure to that of the plant enzyme papain and, therefore, are included in the C1 family of clan CA (cathepsins) (Barrett et al., 2004). All cysteine cathepsins are monomeric single domain enzymes, composed of L (left)- and the R (right)-subdomains, except for Cts C, which is present in the form of a homotetramer (**Figure 1**) (Grzonka et al., 2001; Turk et al., 2012; Löser and Pietzsch, 2015). Following synthesis as inactive preproenzymes (immature), cathepsins become mature after cleavage of the N-terminal signal peptide that occurs in parallel with the N-linked glycosylation within the endoplasmic reticulum (ER). Then, cathepsins are transported to the endosomal/lysosomal compartment using cellular mannose-6-phosphate receptor pathway, where they are activated after removal of the N-terminal propeptide (Chwieralski et al., 2006; Turk et al., 2012). Despite mostly intralysosomal localization, under certain conditions cathepsins can be released from the lysosomes into the cytoplasm, where they perform proapoptotic functions by activating caspases and promoting the release of mitochondrial proapoptotic factors (Chwieralski et al., 2006). Cathepsins cleave a variety of proteins and polypeptides in a relatively unspecific manner. Cts D, E, F, G, K, L, S, and V function as endopeptidases, Cts A, C, and X are exopeptidases, whereas Cts B and H exhibit both exopeptidase and endopeptidase activities (**Table 1**) (Conus and Simon, 2010). The activity of mature cysteine cathepsins is regulated by their endogenous protein inhibitors, such as cystatins, serpins, thyropins and others (Turk et al., 2002).

**Table 1:** Cathepsin family members present in macrophages and macrophage-related cells.

Catalytic type	Cathepsin	Peptidase activity	Expression in macrophages	Reference
Serine	Cathepsin A	Endopeptidase	+	(Jackman et al., 1995)
	Cathepsin G	Endopeptidase	Microglia	(Reeves et al., 2002)
Aspartic	Cathepsin D	Endopeptidase	+	(Rossman et al., 1990)
	Cathepsin E	Endopeptidase	+	(Kakehashi et al., 2007)
Cysteine	Cathepsin B	Carboxydipeptidase, Endopeptidase	+	(Rodriguez-Franco et al., 2012)
	Cathepsin C (J, DPPI)	Aminodipeptidase	+	(Alam et al., 2019)
	Cathepsin F	Endopeptidase	+	(Shi et al., 2000)
	Cathepsin H	Aminopeptidase, Endopeptidase	+	(Woischnik et al., 2008)
	Cathepsin K (O2)	Endopeptidase	Bone‐resorbing macrophages, epithelioid cells, multinucleated giant cells	(Bühling et al., 2001)
	Cathepsin L	Endopeptidase	+	(Beers et al., 2003)
	Cathepsin O	Unknown	MDMs	(Shi et al., 1995)
	Cathepsin S	Endopeptidase	+	(Liuzzo et al., 1999)
	Cathepsin V (L2)	Endopeptidase	Activated MDMs	(Yasuda et al., 2004)
	Cathepsin W (lymphopain)	Unknown	Activated macrophages	(Pires et al., 2016)
	Cathepsin X (P, Y or Z)	Carboxymonopeptidase	+	(Obermajer et al., 2008)

MDMs, monocyte–derived macrophages; (+), ubiquitous expression in macrophages.

Cathepsins are generally widely distributed in cells and tissues in humans and animals, and their level of expression, activity and ratio varies and depends mainly on the location within the cell. While most cathepsins are common in certain cell types, some of them play a specific role only in particular cell types (Zavasnik-Bergant and Turk, 2007). Because macrophages are one of the first cells of the immune system to encounter bacteria, the presence of a specific repertoire of cathepsins enables them to perform efficient innate and adaptive antibacterial functions. Cathepsins can directly interact and participate in killing of invading bacteria, as well as contribute to stimulation of protective microbial-specific immune response through regulation of bacterial antigen processing and presentation. On the other hand, bacteria can influence cathepsin expression and proteolytic activity to favor their own intracellular survival in macrophages and to inhibit the development of a specific immune response. Further, we discuss recent understanding on how bacteria interact with cathepsin functions in macrophages and how dysregulation of expression, ratio and activity of these enzymes participate in the pathogenesis of bacterial infections.

## Cathepsins and Their Presence in Macrophages

Macrophages express a wide range of cathepsin genes and proteins, but often the level of enzyme expression and activity depends on the subtype and activation status of a macrophage (Abd-Elrahman et al., 2016). In general, macrophages have the highest cathepsin expression and all serine, aspartic and cysteine peptidases have been identified in different subtypes of macrophages (**Table 1**). The serine protease, Cts A, is expressed primarily in platelets (Jackman et al., 1990; Ostrowska, 1997), fibroblasts (Satake et al., 1994), cells of the testis and epididymis (Luedtke et al., 2000), human B cells, both subsets of myeloid DCs (mDC1 and mDC2), plasmacytoid DCs (Reich et al., 2010), as well as in human alveolar macrophages (Jackman et al., 1995) and established murine RAW 264.7 macrophage cell line (Chen et al., 2018). Another serine protease, Cts G, is predominantly expressed in azurophilic granules of neutrophils and, to a lesser extent, in B cells, myeloid DCs, plasmacytoid DCs, and cells of the monocyte/macrophage lineage, including primary human monocytes and murine microglia, the latter of which are macrophage-related cells of the central nerve system (Reeves et al., 2002; Burster et al., 2010). The expression and activity of Cts G was reported to be down-regulated in a THP-1 monocytic cell line after differentiation into adherent macrophages by exposure to lipopolysaccharide (LPS) endotoxin. Additionally, Cts G mRNA was absent in macrophages isolated from bronchoalveolar lavages (BAL) and normal blood (Rivera-Marrero et al., 2004). Meanwhile, strong up-regulation of Cts G mRNA level was observed in alveolar macrophages infected with *Mycobacterium bovis* bacillus Calmette-Guérin (BCG) in *in vivo* conditions (Srivastava et al., 2006). The aspartic proteases, Cts D and E, are predominantly distributed in endosomal and/or lysosomal compartments in APCs, such as macrophages (Sakai et al., 1989; Rossman et al., 1990; Kakehashi et al., 2007), DCs (Chain et al., 2005; Kakehashi et al., 2007; Nakken et al., 2011), B cells (Bever et al., 1989; Bennett et al., 1992; Sealy et al., 1996) and microglia (Sastradipura et al., 1998; Nishioku et al., 2002; Kim et al., 2007). In macrophages, Cts D expression is highly differentiation-dependent and was shown to be increased upon maturation of monocytes into macrophages (Sintiprungrat et al., 2010). Macrophages also show the expression of cysteine cathepsins that appear ubiquitously in human cells, including Cts B (Rodriguez-Franco et al., 2012), C (also known as Cts J, dipeptidyl peptidase I or DPPI) (Alam et al., 2019), F (Shi et al., 2000), H (Woischnik et al., 2008) and L (Beers et al., 2003). However, their abundance, with the exception of Cts F, often increases in activated cells (Reddy et al., 1995; Pires et al., 2016). Despite the presence of *Ctso* mRNA in 15-day-old cultures of monocyte-derived macrophages, this transcript was absent in human alveolar macrophages (Shi et al., 1995). Cysteine Cts K (also termed O2) is present in specifically differentiated phenotypes of macrophages located in different anatomical sites, including bone‐resorbing macrophages, and epithelioid cells and multinucleated giant cells (MGCs) of soft tissues. MGCs are probably generated locally during inflammation by fusion of macrophages and epithelioid cells, which are highly stimulated macrophages (Bühling et al., 2001). *Ctsk* mRNA expression was also found in monocyte-derived macrophages (MDMs) after 6 or 12 days of differentiation and Cts K protein was detected in culture supernatants of macrophages (Yasuda et al., 2004). Cysteine Cts S is preferentially expressed in macrophages and microglia, however upon phagocyte activation there is a decrease in cellular Cts S activity and protein content accompanied by an increase in secreted Cts S activity (Liuzzo et al., 1999). Cts S is also present in other APCs, including DCs and B lymphocytes (Riese et al., 1996). Meanwhile, Cts V (also known as L2), which is closely related to Cts L, is mostly confined to the thymus and testis but was also found to be expressed in activated MDMs (Yasuda et al., 2004). Lysosomal cysteine Cts X (also termed P, Y, or Z) is found predominantly in immune cells of the myeloid lineage, including monocytes, macrophages and DCs (Obermajer et al., 2008). In turn, Cts W (lymphopain) is found almost exclusively in cytotoxic cells, i.e., natural killer (NK) cells and CD8^+^ T lymphocytes (Wex et al., 2001), however, single-cell RNA-sequencing analysis revealed the presence of *Ctsw* gene in one of four newly identified human monocyte subpopulations, termed Mono4 that resembles natural killer dendritic cells (NKDCs) based on the signature profile of cytotoxic genes (Villani et al., 2017). Additionally, *Ctsw* mRNA was found to be elevated in activated macrophages (Pires et al., 2016).

Cathepsin proteases can be distinctively expressed during differentiation of macrophages into two main functional phenotypes, named M1 for classical activation phenotype and M2 for alternative activation phenotype. M1/M2 macrophage division is based on the character of the immune response they induce. M1 macrophages, induced by interferon (IFN)-γ and/or LPS, produce high amounts of nitric oxide (NO), ROS and pro-inflammatory cytokines, including tumor necrosis factor (TNF)-α, interleukin (IL)-1β, IL-6, and IL-12, therefore are competent to mount a strong response against intracellular bacteria. Conversely, M2 macrophages can be generated through IL-4 or IL-13 stimulation and secrete high amounts of anti-inflammatory cytokines, such as IL-10 and transforming growth factor (TGF)-β, therefore are not effective in bacteria elimination (Benoit et al., 2008). Stimulation of resting M0 macrophages with IFN-γ results in generation of activated M1 macrophages that exhibit a global up-regulation of cathepsin mRNAs, such as Cts B, C, E, G, H O, S, V, W, and Z, with the exception of mRNA for Cts F, L, and K, where Cts F mRNA is the most strongly down-regulated after IFN-γ treatment (Pires et al., 2016). Additionally, mRNA expression and activity of key cathepsins, i.e., Cts B, L, and S, regulating functions of macrophages, increase significantly in M2 macrophages (Salpeter et al., 2015). One study identified cathepsin genes, which when inhibited or knocked-down cause a shift in phenotype from M1 to M2 macrophages. For example, *Ctsc* is up- and down-regulated in M1 and M2 macrophages, respectively, whereas its deficiency leads to combined M2 (*in vitro*) and Th2 (*in vivo*) polarization (Herías et al., 2015). Treatment of macrophages with an active monomer of Cts C facilitates their polarization toward M1 phenotype through focal adhesion kinase (FAK)-induced p38 mitogen-activated protein kinase (MAPK)/nuclear factor (NF)-κB signaling pathway activation (Alam et al., 2019). Apparently, gut macrophages can be polarized toward M2 phenotype by Cts K secreted through the toll-like receptor (TLR)4 signaling initiated by gut-microbiota (Li et al., 2019). This diverse representation of cathepsins underscores their various roles as regulators of macrophage differentiation and function and possibly influences the outcome of infectious and other diseases.

## Role of Cathepsins in the Function of Macrophages During Homeostasis and Bacterial Infections

Involvement of individual cysteine cathepsins in many bacterial infections is now an established fact, and it points to their potential regulatory role in both innate and acquired antibacterial immune responses. In particular, cathepsin activity is fundamental to the effectiveness of macrophages in recognizing, engulfing, killing, and processing antigens of infecting bacteria, thus facilitating rapid elimination of the intruder and induction of long-term immunity. Accordingly, better understanding of how cathepsins function, their localization, and properties primarily in specialized phagocytic immune cells is imperative for establishing new mechanisms for rational design of therapeutic interventions.

## Cathepsins and innate immune functions of macrophages

### TLR Signaling and Cytokine Production

**Figure 2:**
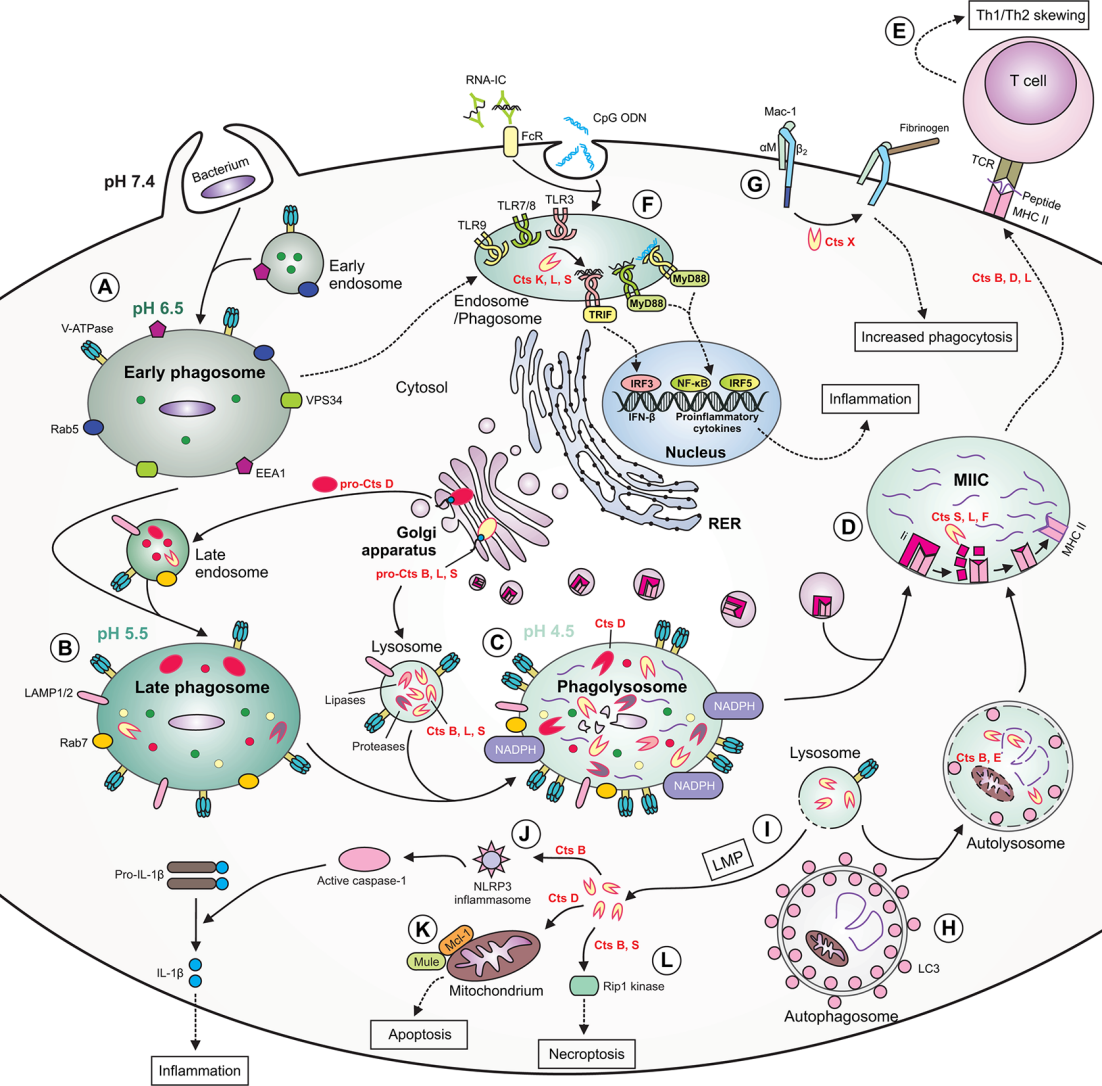
The role of cathepsins in macrophage functions during bacterial infection. **(A)** During phagocytosis of bacteria, the early phagosome quickly develops due to fusion with early endosomes. The early phagosome contains the following markers: early endosome antigen 1 (EEA1), small GTPase Rab5, vacuolar protein-sorting 34 (VPS34) and V-ATPase, the latter which ensures a slightly acidic pH. **(B)** The late phagosome is more acidic and contains Rab7, lysosomal-associated membrane proteins (LAMPs) as well as cathepsins. Some cathepsins are already active, whereas others [e.g., cathepsin D (Cts D)] are present as procathepsins. **(C)** Following fusion of late endosome with lysosome, the phagolysosome with acidic pH is formed. The phagolysosome contains NADPH oxidase responsible for generation of reactive oxygen species (ROS), and many active cathepsins, including Cts B, D, L, and S and other enzymes with proteolytic or lipolytic activities. In this compartment, cathepsins participate in direct killing of bacteria, which are degraded into peptides. **(D)** Major histocompatibility complex (MHC) class II molecules are transported from the ER *via* Golgi complex to a specialized acidic compartment called MHC class II compartment (MIIC). During MHC II trafficking, the invariant chain (Ii) is cleaved by cathepsins (e.g., S, L, and F) leaving CLIP fragment in the MHC peptide binding cleft. In the MIIC, the MHC class II*-*associated invariant chain peptide (CLIP) is released and MHC II molecules can finally bind bacterial peptide, travel to the cell surface and present it to T cells. **(E)** Cts B, D and L may participate in skewing the T helper (Th)1/Th2 immune response. **(F)** Cathepsins, including Cts K, L and S participate in processing of endolysosomal Toll-like receptors (TLRs) and formation of their functional variants, capable of binding ligands. After activation of TIR domain-containing adaptor protein-inducing interferon (IFN) β (TRIF) and myeloid differentiation factor *88*(MyD88), the activation signal is transmitted leading to gene transcription and the synthesis of type I IFNs or proinflammatory cytokines occurs. **(G)** Cts X cleaves β2 subunit of the Mac-1 integrin receptor, enhancing phagocytosis and fibrinogen binding. **(H)** During autophagy, cathepsins participate in degradation of autophagic material within the autolysosome (formed after fusion of autophagosome with lysosome), therefore providing bacterial peptides for MHC II antigen presentation pathway. **(I)** Due to lysosomal membrane permeabilization (LMP), lysosomal enzymes, including cathepsins, may leak into the cytosol. **(J)** Within the cytosol cathepsins (e.g., Cts B) may participate in NLR family pyrin domain containing 3 (NLRP3) inflammasome generation leading to activation of caspase 1, which eventually converts inactive pro-interleukin (IL)-1β into active IL-1β. **(K)** Cytosolic Cts D enhances apoptosis by promoting degradation of myeloid cell leukemia factor 1 (Mcl-1) *via* increased Mule-mediated ubiquitination. (**L**) Cts B and S may promote pyroptosis by cleavage of Rip1 kinase. RNA-IC, RNA immune complex; CpG ODN, CpG oligodeoxynucleotide; TCR, T cell receptor; FcR, Fc receptor; LC3, microtubule*‐*associated protein 1 light chain 3.

Macrophages are equipped with different types of PRRs localized either on the cell surface, in the cytoplasm or within the membrane of intracellular vesicles, including endosomes, phagosomes and lysosomes. Such positioning of receptors facilitates macrophage recognition of bacteria localized in extra- or intracellular compartments. After recognition by PRRs, a wide range of signaling events occur, ultimately leading to induction of a potent inflammatory response. Cathepsins are engaged in processing membrane PRRs located in the lumen of intracellular endosomes/lysosomes, including TLR3, TLR7/8 and TLR9, which recognize bacterial nucleic acids, i.e., double-stranded RNA (dsRNA), single-stranded RNA (ssRNA) and nonmethylated CpG motifs in DNA, respectively (**Figure 2**) (Roberts et al., 2005; Dalpke and Helm, 2012; Kawashima et al., 2013). For instance, upon synthesis, endogenous TLR3 is transported through the Golgi complex to endosomes, where TLR3 ectodomain is rapidly cleaved by proteases, including Cts B, L, and/or S. In murine RAW 264.7 macrophages, cathepsin cleavage of TLR3 is necessary for signaling in response to some microbial dsRNA, but not to polyinosinic-polycytidylic acid [poly(I:C)], a synthetic dsRNA analog (Qi et al., 2012). On the other hand, treatment of human U937 macrophages with z-FA-FMK, which inhibits Cts B, L, and S, resulted in impaired TLR3 signaling in response to poly(I:C) (Toscano et al., 2013). Interestingly, TLR3 is expressed at low levels on the surface of macrophages, where it can participate in modulation of TLR3 responses to dsRNA (Murakami et al., 2014), adding a complexity that cathepsins may affect only the intracellular forms of TLR3 or other PRRs. Similarly, TLR7 exists as a cell surface molecule in uncleaved (trafficking from the ER) and cleaved (trafficking from the endolysosomes) forms (Kanno et al., 2015). In this particular case Ewald et al. (2011) demonstrated the inhibitory effect of z-FA-FMK on cathepsin protease cleavage of TLR7, and subsequently inhibiting production of TNF-α in RAW-264.7 cells stimulated with a TLR7 agonist R848. According to Ewald et al. (2011), the proteolysis of TLR3, TLR7, and TLR9 proceeds in a similar manner and is a multistep process. Initial cleavage removes the majority of the ectodomain and is mediated by multiple members of cathepsin family or asparagine endopeptidase (AEP, also known as legumain), whereas the second step requires exclusively cathepsins and is needed for N-terminal TLR trimming and generation of the mature form of the cleaved receptors. In the absence of AEP activity, both initial processing and trimming of the processed TLR9 are entirely cathepsin-dependent in macrophages. In contrast, the fully mature form of TLR9 cannot be formed, when cathepsin activity is block due to impairment of cleaved receptor trimming (Ewald et al., 2011).

The link between cathepsins and TLR signaling in macrophages is bilateral, because, on the one hand, some TLRs require cleavage by cathepsins for proper signal transduction, but on the other, stimulation of TLR signaling affects the activity of some cathepsins. Treatment of multiple murine macrophage cell lines with LPS, a TLR4 agonist, increases the activity of Cts B, L, and S, whereas stimulation with peptidoglycan (PGN), a TLR2 agonist, and Poly(I:C), a TLR3 agonist, enhances proteolytic activity especially of Cts L and Cts S. Therefore, increased Cts L and S activities involve engagement of either MyD88-dependent or -independent signaling pathways, whereas enhanced Cts B activity involves only MyD88-dependent pathway. However, such regulation of cathepsin activity does not result from the direct TLR signaling, but from the pro-inflammatory cytokines produced during the response of macrophages to TLR agonist treatment. TNF-α and IL-1β were the primary regulators of Cts L and S activities in macrophages (Creasy and McCoy, 2011). IFN-β and IFN-γ were also shown to upregulate cathepsin activity in macrophages (Lah et al., 1995; Creasy and McCoy, 2011).

Cts E also regulates pro-inflammatory response of macrophages since peritoneal macrophages derived from *Ctse^–/–^* mice exhibited reduced production of IL-6 and TNF-α in response to bacterial ligand stimulation, including PGN (TLR2 ligand), LPS (TLR4 ligand), and macrophage-activating lipopeptide 2 (MALP2; TLR6 ligand) compared to wild-type (WT) cells (Tsukuba et al., 2006). Moreover, *Ctse^–/–^* macrophages had decreased surface expression levels of TLR2 and TLR4, despite comparable total cellular levels of these TLRs in WT cells, suggesting that during Cts E deficiency there are defects in transport of these receptors to the cell surface, most probably due to elevated lysosomal pH. For that reason, Cts E-deficient macrophages showed decreased bactericidal activity toward *Staphylococcus aureus*, and *Ctse^–/–^* mice were highly susceptible to infection with Gram-positive *S. aureus* as well as Gram-negative *Porphyromonas gingivalis* compared to WT mice (Tsukuba et al., 2006).

Besides Cts E, Cts K is also involved in IL-6 production by macrophages in response to *S. aureus* (Müller et al., 2014). Moreover, Ha et al. (2008) demonstrated that in response to the bacterial LPS there is a posttranslational process of TNF-α in macrophages, in which extralysosomal Cts B functions as an endopeptidase at neutral pH and regulates the trafficking of vesicles containing TNF-*α* to the plasma membrane through transcriptional or posttranslational regulation of the soluble N-ethylmaleimide-sensitive factor attachment protein receptor (SNARE) proteins, which are the major elements of the intracellular machinery engaged in targeted membrane delivery.

### Phagocytosis

Phagocytosis is the first step of the bactericidal activity of macrophages and is regulated by endolysosomal cysteine cathepsins due to the close relationship between the phagocytic and the lysosomal pathways (Müller et al., 2014). Cathepsins act optimally in reducing and slightly acidic environment, and such a condition is created within the phagolysosome (Sanman et al., 2016). Bacteria are degraded in the phagolysosome not only through oxidative attack, but by nonoxidative killing too, in which cathepsins are involved directly (**Figure 2**). Efficient phagocytosis and killing of *S. aureus* in macrophages is driven by cysteine Cts L, the major endoprotease contributing to the nonoxidative killing pathway. (Müller et al., 2014). In alveolar macrophages of mice infected with *Mycobacterium bovis* BCG, Cts G is strongly upregulated and, together with neutrophil elastase (NE), participates in effective elimination of the engulfed pathogens. It is highly likely that Cts G and NE offer the best proteolytic activity under *in vivo* conditions, because engulfed mycobacteria induce the arrest of phagosome maturation and acidification in macrophages, but Cts G and NE are neutral serine proteases and so can optimally digested the engulfed bacteria at neutral pH (Steinwede et al., 2012).

Cathepsins also regulate the activation of β2 integrin receptors that initiate a variety of macrophage responses associated with phagocytosis, including cell adhesion, migration, respiratory burst and degranulation. Cts X cleaves four C-terminal amino acids within the β2 subunit of the β2 integrin receptor Mac-1 (CD11b/CD18), resulting in the activation of the receptor and achieving two goals, increase in phagocytosis and enhancement of macrophage adhesion to fibrinogen (**Figure 2**) (Obermajer et al., 2006; Kos et al., 2009). Extracellular Cts S influences macrophage and monocyte transmigration through the basal membrane of endothelium (Sukhova et al., 2003).

### Apoptosis/Autophagy

Macrophages apparently can die through lysosome-mediated apoptosis. In this pathway of cell programmed death, certain cathepsins can be released from the lysosomes into the cytosol, where they initiate the apoptotic cascade upstream of mitochondria (Guicciardi et al., 2004). Subsequent clearance of apoptotic cells occurs through their phagocytosis and delivery to endolysosomes for destruction in phagocytic cells. For instance, macrophages infected with *Streptococcus pneumoniae* undergo apoptosis followed by efficient phagocytosis and successful bacterial killing (Dockrell et al., 2001). Upon enclosure of *S. pneumoniae* into phagolysosomes activity of Cts D increases accompanied by its translocation to the cytosol resulting from lysosomal membrane permeabilization (LMP) just before activation of the mitochondrial pathway of apoptosis (Bewley et al., 2011a; Bewley et al., 2011b). Within the cytosol, active Cts D enhances binding of the anti-apoptotic protein Mcl-1 to its ubiquitin ligase, Mule, which mediates degradation of Mcl-1 (**Figure 2**) (Bewley et al., 2011a). Proteomic studies revealed that Cts D regulates multiple proteins engaged in the mitochondrial pathway of macrophage apoptosis, facilitating intracellular killing of *S. pneumoniae* (Bewley et al., 2011b). It is worth mentioning that in addition to aspartic Cts D, cysteine Cts B, C, F, H, K, L, O, S, V, W, and X also function as effectors of lysosomal cell death downstream of LMP in different immune and non-immune cells (Mrschtik and Ryan, 2015; Yadati et al., 2020).

As an integral component of autophagy cathepsins have a prominent role in degradation of autophagic material (**Figure 2**) (Uchiyama, 2001). In macrophages, several studies revealed a link between bacterial infection and activation of autophagy, which can act as a defense mechanism participating in degradation of invading pathogens in a lysosome-dependent manner (Vural and Kehrl, 2014). Tsukuba et al. (2013) have demonstrated that macrophages with Cts E deficiency exhibit abnormalities in autophagy process, manifested by altered autophagy-related signaling pathways and inhibition of autophagosome-lysosome fusion. Such impaired autophagic flux is accompanied by accumulation of aberrant mitochondria and increased oxidative stress in *Ctse^–/–^* macrophages (Tsukuba et al., 2013). This mechanism may partially explain increased susceptibility of *Ctse* ^−/−^ mice to bacterial infection with *S. aureus* or *P. gingivalis* (Tsukuba et al., 2006). Meanwhile, the level of Cts L in human monocyte-derived macrophages (HMDM) is up-regulated by peroxisome proliferator-activated receptor (PPAR) γ, which is a transcription factor engaged in bacterial-induced inflammation, and such activation of Cts L inhibits autophagy and favors apoptosis of these cells. It is suggested that the promotion of macrophage apoptosis *via* PPARγ-induced Cts L may inhibit atherosclerosis progression, because phagocytic clearance of apoptotic macrophages is very effective especially in early atherosclerotic lesions (Mahmood et al., 2011). Currently, it is strongly suggested that some bacterial agents may contribute to the development of atherosclerosis (Ma and Li, 2018).

Besides apoptosis and autophagy, cathepsins in macrophages play a role in necroptosis, a specific form of programmed necrosis. Cts B and S can directly cleave receptor interacting protein kinase-1 (Rip1), which is a key necroptotic kinase (**Figure 2**). Cathepsin-mediated cleavage of Rip1 kinase promotes macrophage survival and perpetuates their function within inflammatory sites (McComb et al., 2014).

## Cathepsins and Macrophage Contribution to Adaptive Immune Response

### Antigen Processing and Presentation

The key role of cathepsins in antigen processing and presentation is fundamental to the development of effective adaptive immunity. Through phagocytosis cathepsins participate in degradation of exogenous antigens into smaller peptides that are then loaded into appropriate MHC II molecules allowing formation of MHC-peptide complexes, which are subsequently presented to CD4^+^ T cells (Conus and Simon, 2010). Aspartate Cts D and E and cysteine Cts B, L and S are involved in this process (Zhang et al., 2000). Additionally, Cts L, S and F are implicated in peptide loading in macrophages by processing out the invariant chain (Ii) linked to MHC class molecules (**Figure 2**) (Shi et al., 2000; Hsieh et al., 2002). Cts L and S predominate protease processing of Ii, however, in the absence of those cathepsins in macrophages, Cts F effectively degrades Ii (Shi et al., 2000). Moreover, during differentiation of monocytes into macrophages, Cts A forms a complex with and activates lysosomal sialidase (neuraminidase) Neu1 and relocates together with Neu1 from the lysosomes to the cell surface, where it participates in antigen presentation (Liang et al., 2006). Another aspartic proteinase, Cts E, is also essential for class II antigen presentation in macrophages, since *Ctse* ^−/−^ macrophages show markedly decreased ability to present intact OVA and OVA-derived antigenic peptide (266–281) to cognate T cells (Kakehashi et al., 2007). Furthermore, Cts S is important in antigen presentation by CD1^+^ macrophages, because *Ctss* ^−/−^ mice exhibit dysfunctional CD1-restricted antigen presentation (Riese et al., 1996; Sukhova et al., 2003).

### Activation of the Th Immune Response

The development of functional subsets of CD4^+^ T cells appear to benefit from the influence of cathepsin proteases. Immunization of BALB/c mice with OVA adsorbed to alum generated weaker Th1 and Th2 responses after treatment with Cts D inhibitor (pepstatin A), whereas mice treated with Cts B inhibitor (CA074) switched from the Th2-type into the Th1-type response induced by OVA (Zhang et al., 2000). Therefore, some cathepsins may create antigenic peptide/motifs that favor development of polarized Th immune responses (**Figure 2**). A good example of this phenomenon is the experimental model of leishmaniasis where susceptible BALB/c mice treated with Cts B inhibitor (CA074) acquired resistance to infection with *Lieshmania major* characterized by the shift from Th2 to Th1 immune response, suggesting that Cts B preferentially activates a Th2 response (Maekawa et al., 1998). Conversely, treatment of *L. major*-infected BALB/c mice with Cts L inhibitor (CLIK148) suppressed the Th1 response and enhanced a Th2 response, suggesting that Cts L is crucial in stimulation of a Th1 response following infection with the *Lieshmania* parasite (Tianqian et al., 2001; Onishi et al., 2004). Since DCs are professional APCs with the most potent activity in priming and polarizing naïve T cells toward different Th subsets, it is possible that cathepsin deficiency-mediated shift between Th1 and Th2 responses is largely dependent on the functioning of DCs in such conditions. However, the missing knowledge gap toward understanding of the mechanisms involved is that there are no data indicating the direct participation of cathepsins in macrophage-driven polarization of the Th immune response.

## Cathepsins as Antibacterial Agents and Targets of Bacterial Modulation in Macrophages

As discussed earlier, cathepsins regulate many innate antibacterial functions of macrophages including those that support induction of adaptive antibacterial responses. Consequently, many bacterial pathogens have evolved multiple strategies to modulate cathepsin availability and functionality in macrophages. These strategies include, but are not limited to, prevention of phagosome-lysosome fusion, alteration in endosomal/phagolysosomal pH, exclusion of cathepsins from the bacteria-containing endosomes, inhibition of recruitment of cathepsins into the phagolysosome, modulation of cathepsin gene and protein expression, alteration of processing and maturation of cathepsins, and down-regulation of cathepsin activity. However, in many cases, cathepsin-modulation strategies depend on the lifestyle of bacteria, whether they are primarily extracellular or intracellular pathogens or whether they lead a dual lifestyle as extracellular/intracellular pathogens. Farther, we discuss cathepsin antibacterial significance as well as bacterial mechanisms for controlling cathepsin activity and functions in macrophages as they relate to individual extracellular, facultative intracellular and obligate intracellular bacterial species, which are clinically important especially for human health.

## Extracellular Bacteria

### *Streptococcus pneumoniae*

Gram-positive *S. pneumoniae* is a causative agent of bacterial pneumonia, meningitis, acute otitis media, acute sinusitis and bacteremia. Aspartic Cts D plays an important role in response of macrophages against *S. pneumoniae* infection (Bewley et al., 2011a). Once Cts D is activated and released into the cytosol, it induces apoptosis through reduction of the Mcl-1 level. The Cts D-dependent induction of apoptosis in macrophages provides a late phase of killing of pneumococci in *in vitro* conditions. Apparently, Cts D activation regulates multiple signaling pathways engaged in mitochondria-dependent apoptosis in macrophages, leading to intracellular killing of *S. pneumoniae* (Bewley et al., 2011b). Under *in vivo* conditions, the protective role of Cts D against pneumococcal infection was demonstrated using *Ctsd^−/−^* mice in which reduced apoptosis of alveolar macrophages was evident accompanied by decreased clearance of pneumococci in the mouse lung (Bewley et al., 2011a). Cat D cleaves streptococcal virulence factor, pneumolysin (PLY) at two sites: between Trp-435 and Trp-436 residues and in residues 361–366 in the D4 domain, rendering the protein void of its biological function (Carrasco-Marín et al., 2009).

Serine protease, Cts G is implicated in lung-protective immunity against focal pneumonia induced by low virulence serotype 19 *S. pneumoniae* in mice. Deficiency of Cts G in mice increases bacterial loads in the lung, causes severe respiratory distress and progressive mortality following infection with *S. pneumoniae.* According to Hahn et al. (2011), increased susceptibility and lung tissue injury was even more progressive in infected double knockout mice, lacking Cts G and serine protease NE. However, the primary cellular source of Cts G and NE are not macrophages, but neutrophils, because specific depletion of neutrophils resulted in complete loss of alveolar Cts G and NE bioavailability in mice infected with *S. pneumoniae*, which was accompanied by uncontrolled outgrowth of bacteria in distal air spaces (Hahn et al., 2011). The bactericidal activity of purified human Cts G and NE against *S. pneumoniae* was also demonstrated in *in vitro* conditions (Standish and Weiser, 2009).

Many pathogens have evolved their own means of defense, and so have bacteria against cathepsins. The rat model of acute otitis media, an inflammatory disease of the middle ear, often caused by infection with pneumococci exemplifies such occurrence. *S. pneumoniae* infection resulted in down-regulation of *Ctsk* and *Ctsl* and dramatic up-regulation of *Ctsb* mRNAs in the middle ear mucosa at 12 and 48 hours post infection (hpi). These results suggest disruption of Cts K and Cts L protein synthesis and possibly functions, whereas Cts B may play a role in acute otitis media pathogenesis during *S pneumoniae* infection (Li‐Korotky et al., 2004). Although macrophages are a major cellular component of human middle ear effusions, S. *pneumoniae* types 14 and 19F happen to be quite resistant to phagocytosis by macrophages, and it is likely the reason they are associated with the highest relapse frequency in cases of acute otitis media, (Bakaletz et al., 1987). Probably the imbalance in cathepsin expression influences macrophage functions and innate immune properties, what, in turn, may contribute to middle ear effusion by a sustained release of proinflammatory cytokines or immune injury (Hannaford et al., 2012). Therefore, further studies are needed to elucidate the role of cathepsins in the functioning of macrophages during acute otitis media. The protective role of cathepsins, especially Cts B, during *S. pneumoniae* infection was documented using the z-FA-FMK inhibitor in a mouse model of intranasal pneumococcal infection. *In vivo* administration of z-FA-FMK to infected animals resulted in increased bacterial growth in lungs and blood, compared with controls, suggesting it partially engagement in the control of pneumococcal infection (Lawrence et al., 2006).

### *Helicobacter pylori*

Gram-negative *H. pylori* is a major human pathogen responsible for chronic gastritis, and infection with this bacterium significantly increases the risk of developing gastric ulcer, duodenal ulcer, gastric cancer (adenocarcinoma), and mucosa-associated lymphoid tissue (MALT) lymphoma. During *H. pylori* infection, there is a persistent infiltration of macrophages and neutrophils in the gastric mucosa, where these phagocytes contribute to development of gastric inflammation and possibly gastric carcinogenesis (Xia et al., 2004; Fu et al., 2016). Patients suffering from *H. pylori-*induced gastritis express significantly more Cts X at both mRNA and protein level than *H. pylori*-negative patients. Additionally, up-regulated expression of Cts X was observed in gastric cancer compared to non-neoplastic mucosa. Macrophages were found to be a major source of Cts X in the mucosal stroma and in glands of the antral mucosa, as well as in the gastric cancer (Krueger et al., 2005). Up-regulation of Cts X in macrophages during infection with *H. pylori* is dependent on the presence of a pathogenicity island (PAI) encoding the type IV secretion system (T4SS) and virulence factor CagA (cytotoxin-associated gene A), and proinflammatory cytokine production. The PAI-or CagA-positive *H. pylori* strains induced up-regulation of Cts X mRNA expression in THP-1 or U937 macrophage-like cells, respectively, through elevation of TNF-α secretion, leading to increased expression of Cts X in macrophages (Krueger et al., 2005; Krueger et al., 2009). Overexpression of Cts X in macrophages stimulated by *H. pylori*-induced cytokines *via* activation of extracellular signal-regulated kinase (ERK)1/2 signaling pathway (Krueger et al., 2009).

Cts X co-localizes with and probably activates Mac-1 integrin receptor within the membrane of THP-1 macrophage-like cells after exposure to antigens of *H. pylori* therapy-resistant strains (Obermajer et al., 2009). Interestingly, such translocation of Cts X was associated with elevated capacity of THP-1 cells to stimulate proliferation of peripheral blood mononuclear cells (PBMC); however, inhibition of Cts X with neutralizing 2F12 monoclonal antibody (mAb) further enhanced macrophage ability to stimulate PBMC proliferation and to form multicellular clusters with PBMC (Obermajer et al., 2009). These results indicate that membrane expression of Cts X may be responsible for inadequate immune response against *H. pylori*, what correlates with the inability to eradicate the infection using standard antibiotic therapy. It is possible that these events are responsible for persistence of chronic infection *in vivo* (Obermajer et al., 2009). Thus, inhibition of Cts X seems to be beneficial to the enhancement of the immune response necessary for eradication of *H. pylori* infection. Furthermore, it has been documented that inhibition of Cts X in THP-1 cells treated with *H. pylori* strains resistant to clarithromycin increases surface expression of TLR4 and prevents its cellular internalization, ultimately decreasing production of cytokines IL-1β, IL-8, IL-10, and IL-6. These data confirm that Cts X localization and activity may impact the efficacy of the immune response during *H. pylori* infection and may partially explain the link between lower immunogenicity and eradication failure of clarithromycin-resistant strains of *H. pylori* (Skvarc et al., 2013).

It has been additionally suggested that during chronic *H. pylori* gastritis Cts X may increase invasiveness of tumor cells by proteolytic cleavage of cellular proteins responsible for cell proliferation and migration (Krueger et al., 2005). On the contrary, further study supports the hypothesis for a protective role of Cts X in metaplastic differentiation, since *Ctsx^–/–^* mice showed higher level of infiltrating macrophages, enhanced epithelial proliferation and more severe spasmolytic polypeptide expressing metaplasia (SPEM), which is associated with progression of gastric cancer, than WT animals (Krueger et al., 2013). Therefore, it will be intriguing to elucidate whether Cts X plays a protective versus pathogenic role in cancer development during chronic *H. pylori* infection.

Another cathepsin-dependent mechanism by which *H. pylori* ensures persistent infection in gastric mucosa has been described, in which abolition of Cts C expression weakens the activation of neutrophils (Liu et al., 2018). Indeed, in gastric mucosa of human patients and mice infected with *H. pylori* there is a decreased expression of Cts C at both the mRNA and protein levels because of altered expression and secretion of this protease by infected gastric epithelial cells. The pathogen-induced down-regulation of Cts C expression in gastric epithelial cells is achieved through Src-phosphorylated CagA-ERK and CagA-Janus kinase(JAK)/signal transducer and activator of transcription 3 (STAT3) non-phosphorylated pathways, again emphasizing the importance of *H. pylori* virulence factor CagA (cytotoxin-associated gene A). Expression of Cts C in the human gastric mucosa was negatively correlated with pathogen colonization. Moreover, administration of active enzyme decreased gastric bacterial burden in mice. *In vitro* and *in vivo* studies revealed that human neutrophils exhibit increased bactericidal activity in the presence of active Cts C (Liu et al., 2018). Therefore, *H. pylori* manipulation of Cts C represents an immune evasion strategy of the bacterium from neutrophil clearance leading to infection persistence in gastric mucosa.

### *Pseudomonas aeruginosa*

The opportunistic Gram-negative *P. aeruginosa* is the causative agent of a broad spectrum of diseases, including, but not limited to, pneumonia, urinary tract infections, bacteremia, septicemia, and wound infections. These infections are mostly nosocomial and especially in immunocompromised patients, burn patients and cystic fibrosis (CF) patients, the latter of which are extremely susceptible to chronic *P. aeruginosa* infections with a fatal outcome (Bhagirath et al., 2016). During CF lung disease, there is a chronic and unresolved immune response with the predominant presence of neutrophils and macrophages, which are unable to clear the airway compartments from various colonizing and biofilm-forming bacteria, especially *P. aeruginosa*. The intense inflammatory response eventually leads to airway obstruction and bronchiectasis in CF patients (Sedor et al., 2007).

Macrophages in CF demonstrate high plasticity of their phenotype (M1 vs M2), hyperinflammatory potential, altered lysosomal function and decreased phagocytic activity, resulting in impaired capacity for directing the resolution of either infection or inflammation (Bruscia and Bonfield, 2016). *P. aeruginosa* has been shown to evade intracellular killing in macrophages by stimulating the NLRP3 inflammasome and subsequently activating autophagy (Deng et al., 2016). Additionally, proteomics analysis revealed that LasB, an important component of the type II secretions system (T2SS) of *P. aeruginosa*, down-regulates the production of many secreted innate immune components, including C3 and factor B complement molecules as well as Cts B and Cts H in murine alveolar macrophages. This probably contributes to reduced bacterial clearance and increased pathogen virulence (Bastaert et al., 2018). The aspartic protease Cts D may foster a protective role, at least as has been reported during infection of RAW 264.7 macrophages with *P. aeruginosa*. Infected cells upregulated Cts D at both the mRNA and the protein levels, but deficiency of the cathepsin allowed survival of bacteria in macrophages. Therefore, Cts D may directly target bacteria or induce production of proteins with bactericidal activity (Fu et al., 2020). In a murine model of endobronchial inflammation the neutrophil-derived serine protease, Cts G, inhibited clearance of *P. aeruginosa* from the murine lung and subsequently stimulated a greater inflammatory response in the airway (Sedor et al., 2007). Clearly, cathepsins may interfere with airway defense mechanisms, for instance, sputum samples from CF patients whose lungs are colonized by *P. aeruginosa* have higher cathepsin activity but reduced ability to inhibit biofilm formation compared with sputum samples from *P. aeruginosa*-negative CF patients (Rogan et al., 2004). Enzymatically active forms of cysteine cathepsins such as Cts B, H, K, L, and S are not correlated with bacterial colonization, because comparable activities of these enzymes were found in sputum of *P. aeruginosa*-positive and *P. aeruginosa*-negative CF patients, and therefore, they are not suitable markers for this type of infection (Naudin et al., 2011).

## Facultative Intracellular Bacteria

### *Mycobacterium tuberculosis*

*M. tuberculosis*, a poorly Gram-positive bacillus, is the etiological agent of tuberculosis (TB), which is the single leading cause of deaths worldwide, especially among HIV-infected people (Guinn and Rubin, 2017). The most common form of infection affects the lungs (pulmonary TB), but it can also affect lymph nodes, bones, brain, spine or other parts of the body (extrapulmonary TB). The tubercle *bacillus is* a highly effective pathogen that has developed a series of strategies for modulating the immune response of lung macrophages. Within the macrophages, M. *tuberculosis* can survive and even proliferate, since it inhibits phagosome-lysosome fusion, generation of ROS and RNS, induction of autophagy and apoptosis, production of cytokines, and presentation of antigens in the context of MHC molecules (Bhat and Mukhopadhyay, 2015; Ren et al., 2017). The NF-κB–dependent impairment of delivery of lysosomal enzymes to phagosomes is a well-characterized survival mechanism of mycobacteria, which allows them to avoid contact with active cathepsins (Gutierrez et al., 2008). Mycobacteria can also prevent direct digestion by cathepsins in macrophages by escaping from the phagosome into the cytosol, resulting in activation of the inflammasome and subsequent stimulation of Cts B–dependent pyroptosis or pyronecrosis (Welin et al., 2011; Pires et al., 2016; Amaral et al., 2018). Recently, *M. tuberculosis* Rv0297 has been shown to down-regulate Cts D and Rab7 expression in macrophages, suggesting its role in modulation of phagosomal maturation (Sharma et al., 2020). Avoidance of cathepsin-dependent killing by mycobacteria allows them to effectively replicate and spread to neighboring cells.

In addition to avoiding direct contact with cathepsins, *M. tuberculosis* can also modulate the expression of the mRNA or activity of these enzymes. A comparative study using a pathogenic strain *M. tuberculosis* and a non-pathogenic strain *M. smegmatis* revealed that following infection with *M. tuberculosis* mRNAs for the majority of cathepsins and cystatins were down-regulated in M0 (Cts B, C, D, E, G, K, O, S, V, and W and cystatins B, C, D, SA, SN, and E/M) and M1 (Cts B, C, F, K, S, W, and Z and cystatin C) human primary macrophages, in contrast to *M. smegmatis* that induced up-regulation of most cathepsins in both type of cells. The only exception in *M. tuberculosis* infection of M0 and M1 macrophages was Cts L which was up-regulated. Quantitative analysis of cathepsin biosynthesis and enzymatic activity showed that *M. tuberculosis* infection decreases both quantity and activity of Cts B and S, but not L. Such global downregulation of cathepsin expression and activity profoundly decreased pathogen killing and improved its intracellular survival. Pharmacological treatment with a general inhibitor of cysteine cathepsins E-64d, natural cystatin C-based inhibition of Cts B, Cts S, and Cts L or siRNA–mediated gene silencing for Cts B, S, and L, all resulted in significant increase of *M. tuberculosis* survival in primary human macrophages. Additionally, knockdown of Cts B, D, G, L, V, S, W, and Z using a lentivirus-based siRNA approach resulted in increased survival of bacteria within THP1 macrophages, whereas knockdown of Cts F caused increased pathogen killing in these cells (Pires et al., 2016). Overall, cathepsins in macrophages are relevant for the control of *M. tuberculosis* infection, but the pathogen intricately manipulates their expression and activity to further its survival.

A somewhat different result was obtained in murine bone marrow-derived macrophages (BMDMs) infected with pathogenic *M. tuberculosis* or *M. avium*, the latter which causes opportunistic infections in humans and animals (Nepal et al., 2006). In BMDMs both mycobacteria species did not alter activity of Cts B and Cts S, but altered activity of Cts L. The mycobacteria impaired processing of pro-Cts L into the 24kDa two-chain form of active Cts L, suggesting that Cts L does not mature properly in BMDMs infected with mycobacteria (Nepal et al., 2006). Considering that Cts L plays a significant role in processing subsets of antigens that shape the repertoire of MHC class II-associated peptides, this unique evasive mechanism by mycobacteria presumably prevents the generation of protective T cell epitopes in antigen-presenting cells, and/or decreases the rate of class II MHC peptide loading (Hsieh et al., 2002). In support of this observation experimental data derived by Singh et al. (2006) also implicated inhibition of Cts D activation in the impaired processing and presentation of antigens by MHC class II molecules in macrophages infected with virulent strains of mycobacteria. In those studies, bacteria were able to exclude vacuolar proton ATPase (v-ATPase) from phagosomes, preventing acidification (maturation) and breakdown of immature Cts D into the active form. Arrest of phagosome maturation and inhibition of Cts D conversion to active forms in infected macrophages reduced the generation and presentation of immunodominant antigen epitopes to T cells resulting in low IL-2 production (Singh et al., 2006).

Pathogenic species of *Mycobacterium*, including *M. tuberculosis* and *M. bovis*, can also attenuate intracellular trafficking and surface expression of MHC class II molecules on macrophages through post-transcriptional regulation of Cts S expression (Brown et al., 2020), thus promoting their intracellular survival. This regulation may be accomplished by a few factors, including miRNAs. During *M. tuberculosis* infection, miR-106b-5p, which can bind to Cts S mRNA, is strongly up-regulated in macrophages. In contrast, challenge with non-virulent *M. smegmatis* has no effect on Cts S gene expression (Pires et al., 2017). The up-regulation of miR-106b-5p resulted in decreased Cts S activity accompanied by increased intracellular survival of *M. tuberculosis* and reduced expression of human leukocyte antigen (HLA)-DR class II on macrophages, similar to what occurs during silencing of Cts S by siRNA (Pires et al., 2017). Additionally, *Mycobacterium bovis* BCG has been shown to interfere with miR-3619-5p control of Cts S activity in the process of autophagy in THP-1 macrophages (Pawar et al., 2016). Down-regulation of cathepsin S activity and gene expression in human macrophages infected with mycobacteria also depends on IL-10 production (Sendide et al., 2005). Macrophages infected with *M. bovis* BCG produce large amounts of IL-10 concomitant with decreased Cts S activity and reduced translocation of peptide loaded MHC class II complexes to the cell surface. However, these negative effects could be reversed by antibody neutralization of IL-10 or transfection of BCG-infected macrophages with active recombinant Cts S (Sendide et al., 2005). Restoration of surface levels of MHC class II molecules in macrophages could be achieved also by infection of cells with a recombinant BCG strain engineered to express and secrete biologically active human Cts S (Soualhine et al., 2007). Another reason for the impaired trafficking and intracellular retention of MHC class II molecules in BCG-infected macrophages was the intraphagosomal production of urease and subsequent alkalization of endosomes, responsible for MHC class II processing and loading (Sendide et al., 2004). Endosome alkalization therefore may not only affect the Cts S but also the activity of key proteases participating in MHC class II presentation pathway (Baena and Porcelli, 2009).

Conversely, there is an increase in expression of Cts B and/or its activity in THP-1 macrophages and BMDMs infected with mycobacteria (Rivera-Marrero et al., 2004; Amaral et al., 2018). These alterations in Cts B by *M. tuberculosis* are common in lungs of infected mice, infected rabbits or plasma of human patients with active TB, suggesting that there is an association between increased Cts B levels and active TB (Amaral et al., 2018). Interestingly, during *M. tuberculosis* infection of macrophages, mature Cts B is released from the lysosomes into the cytosol, as a consequence of lysosomal destabilization caused by antigens encoded by the mycobacterial genome known as region of difference 1 (RD-1), and secretory mycobacterial antigen ESAT-6, which forms pores in the phagosome membrane. Within the cytosol, Cts B drives NLRP3-inflammasome activation with subsequent production of IL-1β (Amaral et al., 2018). The understanding here is that the major goal of Cts B activation in mycobacteria infection is to induce the maturation of IL-1β, the cytokine that plays a major role in the host protection against *M. tuberculosis* infection (Mayer-Barber et al., 2014).

Pathogenic mycobacteria may also influence the level of Cts G in macrophages. In THP-1 cells the expression and activity of Cts G is down-regulated upon exposure to a virulent strain of *M. tuberculosis* or bacterial LPS. Down-regulation of Cts G expression positively correlates with increased bacterial survival, thus creating an immune evasion mechanism for *M. tuberculosis* (Rivera-Marrero et al., 2004). Similarly, *in vivo* infection of alveolar macrophages with non-virulent *M. bovis* BCG strain initially resulted in down-regulation of *Ctsg* mRNA at 12 h post infection; however, at 3 and 7 day of infection, Cts G was strongly up-regulated, suggesting importance of this protease in the early host defense against mycobacterial infections (Srivastava et al., 2006). However, Cts G-deficient mice could not eliminate *M. bovis* BCG, resulting in increased bacterial loads in the lungs (Steinwede et al., 2012). Neutrophils are the main population of professional phagocytes responsible for delivery of Cts G into the bronchoalveolar space of *M. bovis* BCG-infected mice. It is suggested that Cts G and other proteolytic enzymes may be shuttled into mycobacteria-infected alveolar macrophages together with phagocytosed apoptotic neutrophils (Steinwede et al., 2012). Within macrophages, phagocytosed neutrophil with their granule contents are then transported to the early endosomes and colocalize with mycobacteria, promoting the antimycobacterial activity of macrophages and facilitating the killing of *M. tuberculosis* (Tan et al., 2006; Steinwede et al., 2012). The bacterial protein Rv3364c, secreted by infected macrophages, binds to and inhibits activity of membrane Cts G leading to suppression of activation of caspase-1-dependent apoptosis (Danelishvili et al., 2012). A recent report shows that Cts X, in the presence of the nitric oxide (NO), is involved in rapid killing of pathogenic *Mycobacterium avium* subsp. *hominissuis* in host macrophages, and the virulence factor MAV_4644 serves to protect the pathogen from the killing process (Lewis et al., 2019).

### *Neisseria gonorrhoeae*

*N. gonorrhoeae* is a Gram-negative pathogen responsible for the sexually transmitted disease, gonorrhea with substantial morbidity in humans. This pathogen primarily infects the urogenital tract; however, it may spread from the local site of infection contributing to development of pelvic inflammatory disease, endocarditis, arthritis and dermatitis. Gonococcal infection is characterized by a local inflammatory response driven by a large number of neutrophils and macrophages, especially during acute gonorrhea. *N. gonorrhoeae* is able to survive within macrophages, which are quite important cells involved in the pathogenesis of gonorrhea (Château and Seifert, 2016). This pathogen induces activation of the NLRP3-dependent signaling pathway in THP-1 cells and primary human monocytes, which is required for secretion of mature IL-1β and induction of the cell death *via* pyronecrosis (**Figure 2**). All these processes are dependent on activation of Cts B by the gonococcus, since selective inhibition of this protease with Ca-074-me resulted in reduced NLRP3-mediated IL-1β secretion and pyronecrosis (Duncan et al., 2009). Therefore, modulation of Cts B activity may represent an important bacterial mechanism involved in regulation of inflammatory response and pathogenesis of infections caused by *N. gonorrhoeae*. Cts B targets bacterial penicillin-binding protein 2 (PBP2), which is an essential peptidoglycan transpeptidase engaged in cell division (Shafer et al., 1990; Tomberg et al., 2017). Moreover, the structure of PBP2 and/or intracellular availability may determine the level of gonococcal susceptibility to this cathepsin (Shafer et al., 1990).

On the other hand, lysosomal serine Cts G can directly kill *N. gonorrhoeae* (Shafer et al., 1986; Shafer and Morse, 1987). It does so by degrading porin and colony opacity-associated proteins (Opa) in the *N. gonorrhoeae* outer membrane (Shafer and Morse, 1987). Opa-expressing *N. gonorrhoeae* strains are more sensitive to killing inside primary human neutrophils than Opa-deficient strains. However, bacterial exposure to Cts G resulted in a comparable dose-dependent killing of both Opa-expressing and Opa-deficient strains. The increased susceptibility of Opa-expressing gonococci to neutrophil killing is mediated by CEACAM-dependent triggering of Src family kinase signaling, which promote bacteria trafficking into mature, degradative phagolysosomes where the bacteria are exposed to components with antigonococcal activity, including bactericidal-permeability-increasing protein (BPI) (Johnson et al., 2014).

### *Listeria monocytogenes*

*L. monocytogenes* is a Gram-positive intracellular food-borne pathogen that infects humans and many animal species. It is responsible for listeriosis, affecting mainly pregnant women, their fetuses, and immunocompromised individuals, causing meningoencephalitis, meningitis, septicemia, and brain abscess (Farber and Peterkin, 1991). Early resistance to listeria infection is mediated by IFN-γ production by NK cells and pro-inflammatory response of macrophages, which in turn enhance IFN-γ producing capacity of NK cells (Tripp et al., 1993; Unanue, 1997). Macrophages are generally thought to be the major population of phagocytes responsible for intracellular elimination of *L. monocytogenes*, however the bacterium has evolved many strategies to evade immune defense mechanisms mediated by macrophages (Wang et al., 2017).

Cts D expression increases in macrophages infected with *L. monocytogenes* and plays an important listeriobicidal role in these cells (del Cerro-Vadillo et al., 2006; Fu et al., 2020). RAW 264.7 macrophages up-regulate Cts D expression at both mRNA and protein level during *Listeria* infection. Cts D-deficiency achieved either by treatment with pepstatin A (a specific inhibitor of cathepsin D) or generation of *Ctsd* ^–/–^ cells, results in increase in the number of viable bacteria (Fu et al., 2020). Furthermore, BMDMs of Cts D-deficient mice showed 4- to 5-fold higher replication index of *Listera* than BMDMs of *Ctsd* ^+/+^ mice. Additionally, *Ctsd* ^–/–^ cells contained higher number of freely localized bacteria within the cytosol compared with WT cells. *In vivo* studies underline the importance of Cts D in early resistance to listeriosis, since infection of *Ctsd* ^–/–^ mice resulted in 10-fold increase in bacterial burden in spleen and livers compared to *Ctsd* ^+/+^ littermates (del Cerro-Vadillo et al., 2006). The molecular mechanism by which Cts D influences *L. monocytogenes* virulence was described by Carrasco‐Marín et al. (2009) and involves specific cleavage of listeriolysin O (LLO). LLO, a pore-forming thiol-activated cytolysin, and phosphatidylinositol phospholipase C (PI-PLC), are two major virulence factors of *L. monocytogenes*, allowing the bacterium to escape from the phagosomes to the cytoplasm within host cells, including macrophages (Lauer et al., 2002). Specifically, Cts D-mediated cleavage of LLO occurs between Trp‐491 and Trp‐492 residues in the domain 4(D4) of LLO. Neither Cts D nor Cts L showed any effect on PI‐PLC. Therefore, lysosomal and soluble active Cts D forms that are present abundantly in bacteria phagosomes can cleave LLO monomers and participate in intraphagosomal killing of *L. monocytogenes* (Carrasco‐Marín et al., 2009). In addition to Cts D, also Cts G, derived from human neutrophils, can destroy *Listeria* in *in vitro* conditions (Alford et al., 1990).

### *Staphylococcus aureus*

*S. aureus* is a Gram-positive bacterium causing both community- and hospital-acquired infections with significant morbidity and mortality (Magill et al., 2014a; Magill et al., 2014b). Methicillin-resistant *S. aureus* (MRSA) is responsible for invasive, drug-resistant skin and soft tissue infections contributing to the development of diseases, such as endocarditis, osteomyelitis, or bacteremia (Klevens et al., 2007; Brann et al., 2019). Macrophages are the major cells responsible for clearance of bacteria from infected tissues, however *S. aureus* survives and even replicates inside these cells (Lacoma et al., 2017). After phagocytosis, the *S*. *aureus* containing phagosomes (SaCPs) are formed, which quickly acquire early (Rab5) and then late (LAMP‐1 and Rab7) endosomal markers (Moldovan and Fraunholz, 2018). Nevertheless, SaCPs lack the key lysosomal hydrolases, including beta‐glucuronidase and cathepsin D, suggesting the absence of fully matured phagolysosomes in infected macrophages (Jubrail et al., 2016; Tranchemontagne et al., 2016). More than 60% of phagosomes containing live or heat-killed (HK) highly virulent community-acquired USA300 clone of *S. aureus* co-localized with cathepsin D at 1 hpi, whereas significant reduction of co-localization between live bacteria and cathepsin D was noted at 4 and 8 hpi compared to that observed in HK bacteria-treated macrophages. Additionally, USA300 strain persisted and replicated within the phagosome, and the acidification of this compartment was necessary for bacteria survival. This indicates that live *S. aureus* clone USA300 can actively disturb phagosomal accumulation of lysosomal hydrolyses, limiting pathogen exposure to degradative enzymes (Tranchemontagne et al., 2016). In *in vitro* experimental models such as RAW 264.7 cells, Cts D expression increases during *S. aureus* infection and plays an important role in controlling the growth and viability of this bacterium within macrophages (Fu et al., 2020). The pathogen is degraded in Cts D-positive active lysosomes in primary human macrophages, but still the majority of bacteria survive in late phagosomes (Brann et al., 2019).

During *S. aureus* invasion, the phagolysosomal biogenesis in macrophages is tightly controlled by the proteins belonging to the COMMD (copper metabolism gene MURR1 domain) family, which regulate intracellular trafficking. COMMD10-deficient BMDMs have decreased expression of numerous genes important for the functioning of lysosomes, including Cts B and D, but not K, at 2 and 4 hpi with *S. aureus*. In addition, COMMD10-deficient macrophages infected with *S. aureus*, there is altered exchange of RAB5 to RAB7, peripheral mislocation of LAMP-1 and reduced acidification of phagosomes, all which contribute attenuated bacteria-induced phagolysosomal maturation. This impaired phagolysosomal biogenesis in COMMD10-deficient cells favors the survival of bacteria within macrophages (Shlomo et al., 2019).

Besides Cts D, Cts L is also responsible for nonoxidative killing of *S. aureus* within macrophages, because in the absence of Cts L in primary BMDMs, *S. aureus* can survive intracellularly for at least 3 hpi compared to WT cells, whereas single knockout cells for Cts B, Cts H, Cts K, or Cts Z comparably killed the bacteria as WT macrophages. Although the cysteine cathepsin Cts K is not involved in direct killing, it is critical for efficient production of IL-6 by infected macrophages through the MyD88-dependent TLR signalling (Müller et al., 2014).

In cell types other than macrophages, e.g., neutrophils, lysosomal cysteine cathepsins may indirectly regulate bactericidal and inflammatory activities against *S. aureus.* For instance, Cts C contributes to indirect killing of *S. aureus* in neutrophils through generation of bactericidal proteins or activation of antimicrobial enzymes within the phagolysosomes (Liu et al., 2012). Neutrophil-derived Cts G has limited and delayed bactericidal effect, because *S. aureus* EapH1 inhibits the activity of the enzyme *via* formation of highly complementary, non-covalent complex with this protease that blocks substrate access to the enzyme active site (Herdendorf et al., 2020).

### *Francisella tularensis*

*F. tularensis* is a gram-negative facultative intracellular bacterium that causes tularemia—a potentially fatal infection in humans. This pathogen replicates predominantly in macrophages, but can proliferate in other cell types too (Pechous et al., 2009; Santic et al., 2010). Four subspecies of *F. tularensis* are currently known: *tularensis*, *holarctica*, *mediasiatica*, and *novicida*, wherein subspecies *tularensis* and *holarctica* are mainly responsible for disease in humans (Keim et al., 2007; Santic et al., 2009). Following uptake by macrophages, *F. tularensis* enters a phagosome that acquires minimal amounts of the late endosomal-lysosomal markers, including CD63, LAMP1, and LAMP2, but excludes cathepsin D (Clemens et al., 2004; Asare and Kwaik, 2011). Late endosomal *Francisella*-containing vacuole (FCV) disintegrates within a few hours allowing the pathogen to escape into the cytoplasm, where it replicates. Interestingly, T cells from vaccinated mice can provide a functional assistance in arresting *Francisella* attenuated live vaccine strain (LVS) replication and inhibiting the spread of LVS infection between macrophages in *in vitro* conditions. Co-culture experiments of LVS-infected macrophages with naïve or LVS-immune lymphocytes revealed that LVS-immune T cell control of replication and spread of LVS in macrophages is mediated by a direct effect on the viability of bacteria in the cytoplasm, rather than intracellular trafficking of bacteria into lysosomes for degradation in infected macrophages (Bradford and Elkins, 2020).

Pathomechanisms of tularemia infection have best been studied in mice and rabbits. Removal of Cts B in mice rendered them resistant to infection with *F. novicida* since they had significantly lower bacterial loads in the liver and spleen compared to individuals with normal expression of this protein. Measured at 3 day after infection, Cts B-knockout animals produced less pro-inflammatory cytokines and chemokines in the liver (Qi et al., 2016). Macrophages are the frontline in controlling intracellular growth and dissemination of *Francisella* (Hall et al., 2008) and the lack of Cat B in these cells enhanced their bactericidal activity against the pathogen (Qi et al., 2016). Interestingly, cell structure analysis by transmission electron microscopy revealed that uninfected macrophages lacking cathepsin B contained larger lysosomes and autophagosomes, and accumulated partially digested vesicles in autophagosomes, compared to WT cells. Mechanistically, the increased bactericidal activity against *F. novicida* in macrophages with genetic deletion or pharmacological inhibition of Cat B is a result of upregulated lysosomal biogenesis and autophagy due to downregulation of mechanistic target of rapamycin (mTOR), lysosomal calcium channel TRPML1 and others such as transcription factor EB (TFEB) and phosphorylation of autophagy initiation kinase ULK1 (unc-51-like kinase 1) (Man and Kanneganti, 2016; Qi et al., 2016).

### *Brucella abortus*

*B. abortus* is an intracellular Gram‐negative cocobacillus that survives and replicates within host monocytes and macrophages. This pathogen is the causative agent of brucellosis in humans and livestock. In humans, *B. abortus* evokes fever, endocarditis, arthritis and osteomyelitis; in livestock, it is responsible for abortion and infertility (Pappas et al., 2005; Oliveira et al., 2011). Upon internalization, *Brucella* arrests phagosome maturation between the steps of acidification and phagosome-lysosome fusion and cannot be destroyed within the *Brucella*-containing vacuole (BCV) (Rittig et al., 2001; Celli et al., 2003). In human macrophage-like cell line THP-1, delivery of Cts D to phagosomes occurred simultaneously with the arrival of LAMP-1 and acidification of the lumen of the phagosomes at the early stages of infection (60 min pi) with virulent or HK *B. abortus* 2308. Together with the time progression, the number of Cts D-positive phagosomes containing live *B. abortus* 2308 was significantly reduced when compared to phagosomes with HK bacteria, and after 24 h such vesicles contained multiple brucellae, indicating efficient replication of the pathogen within such compartments (Bellaire et al., 2005). During later stages of infection, BCVs were shown to fuse with lysosomes, what is needed for further maturation of BCVs into an endoplasmic reticulum (ER)-derived organelle, in which bacteria is able to replicate (Bellaire et al., 2005; Starr et al., 2008). Several virulence factors determine *Brucella* survival in macrophages, including zinc-dependent metalloproteinase (ZnMP). Its deletion causes an increase in the co-localization of bacteria with phagosomal Cts D, reducing intracellular replication of the pathogen within RAW 264.7 cells (Gómez et al., 2020). Importantly, *B. abortus* persistence in macrophages is associated with the presence of anti-inflammatory cytokine, IL-10 (Xavier et al., 2013). This cytokine suppresses lysosome-mediated killing of bacteria in macrophages *via* two distinct regulatory mechanisms. Firstly, IL-10 inhibits recruitment of membrane trafficking regulators, including RAB family proteins, LAMP-1, LAMP-2 and cathepsins (Cts A and Cts D), to *B. abortus* phagosomes through a STAT3-independent pathway. Secondly, IL-10 down-regulates the expression of proinflammatory cytokines through activation of the STAT3/SOCS3 (suppressor of cytokine signaling 3) pathway in RAW 264.7 macrophages (Hop et al., 2018).

Although, there are no available data concerning the influence of *Brucella* on cathepsin activity in macrophages and the role of these enzymes during *Brucella* infection, Coria et al. (2016) have demonstrated that unlipidated outer membrane protein 19 (U-Omp19) of *Brucella* partially limits the activity of purified lysosomal proteases, including Cts L, Cts C, Cts B, and papain *in vitro* and also inhibits the digestive capacity of microsomal content derived from murine BMDMs and BMDCs. Additionally, U-Omp19 reduces the ability of macrophages and DCs to degrade extracellular Ag but increases the amount of Ag inside DCs due to inhibition of its intracellular proteolysis within lysosomal compartments. Thus, intracellular half-life of Ag is extended allowing prolongation of intact peptide export to the cytosol, eventually providing long-term Ag cross-presentation and stimulation of Ag-specific CD8^+^ T cell responses *in vivo*.

### *Shigella flexneri*

*S. flexneri*, a Gram-negative enteroinvasive bacteria, is the causative agent of shigellosis – a gastrointestinal disease associated with watery or bloody diarrhea, cramping, and dehydration. *Shigella* infection occurs particularly in young children (under 5 years old) and immunocompromised adults in developing countries and causes significant morbidity and mortality (Kotloff et al., 2013; Kotloff et al., 2018). *Shigella* crosses the intestinal epithelium by transcytosis through microfold cells (M cells), reaching resident macrophages and DCs in the M cell pocket. Bacteria can survive and replicate in macrophages, escape the phagocytic vacuole, and kill the host cell by inducing caspase-1-dependent pyroptotic cell death, allowing subsequent invasion of epithelial cell layer (Schroeder and Hilbi, 2007; Ashida et al., 2014).

Currently, the role of cathepsins during *S. flexneri* replication in macrophages is not known, although the importance of these enzymes in the regulation of the pathogenesis of *Shigella* infections was hghlighted more than 35 years ago. The activity of Cts D in splenocytes of CBA mice infected with *Shigella* strains was variable in the cytoplasmic and lysosomal cell compartments and depended on the virulence of *Shigella* strains. Strong and prolonged activation of Cts D was observed in both compartments after infection with virulent *Shigella* strains, whereas avirulent strains induced only temporary Cts D activity in the lysosomes (Belaia et al., 1984). According to authors, determination of the Cts D activity in splenocytes of animals infected with *Shigella* allows differentiation of virulent and from avirulent strains of the pathogen. Future studies should elucidate the major implications of cathepsins in host defense and pathogenesis of *Shigella* infections.

### *Salmonella enterica* serovar Typhimurium

*S.* Typhimurium is a pathogenic Gram-negative rod bacterium that causes foodborne salmonellosis in humans and a wide range of animal species (McSorley, 2014). Infection of laboratory mice with *S.* Typhimurium results in disseminated infection with some similarities to human disease caused by the human-host restricted *S. enterica* serovars Typhi and, to a lesser extent, Paratyphi (Mathur et al., 2012 *).* Therefore, *S.* Typhimurium infection of mice is widely accepted as an experimental model for human disease (Hilgenberg et al., 2014).

*Salmonella* penetrates the epithelium of the small intestine following oral ingestion, and preferentially infects phagocytes, including macrophages, within lamina propria. *S.* Typhimurium can reside and replicate in macrophages (Salcedo et al., 2001); therefore, these cells are a niche for intracellular survival and from where bacteria can disseminate to distal organs, such as liver and spleen (Vazquez-Torres et al., 1999). To survive in macrophages, the bacteria avoid intracellular killing by preventing lysosome fusion with the modified endosome, known as *Salmonella*-containing vacuole (SCV) (Alpuche-Aranda et al., 1994; Salcedo et al., 2001; Steele-Mortimer, 2008; Gogoi et al., 2019). It is likely that *S.* Typhimurium blocks maturation of its phagosome, because phagosomes isolated from *S.* Typhimurium*-*infected macrophages contained only pro-Cts L and not mature Cts L (Mills and Finlay, 1998), whereas Cts B activity is decreased in macrophages (Sarkar et al., 2017).

Acidification of SCV is necessary for bacteria virulence (Arpaia et al., 2011), so there is a possibility that the *Salmonella* can be digested by endosomal proteases such as cathepsins, which are mostly active at acidic pH values. Therefore, *S.* Typhimurium excludes active cathepsins from the SCV in primary murine macrophages (Hang et al., 2006; Sanman et al., 2016). But other studies have reported that upon initial infection of BMDMs (2 h), the cysteine cathepsins gained access to *S.* Typhimurium in compartments of varied pH (Sanman et al., 2016). Bacteria also induced the increase in lysosomal pH resulting in an overall decline in cysteine cathepsin activity not only in infected but also in a fraction of bystander cells, indicating a mechanism by which *Salmonella* can alter the functionality of nearby uninfected cells (Sanman et al., 2016).

In *S*. Typhimurium infection, cathepsins may not directly target the bacteria but may be involved in triggering pyroptotic cell death of infected macrophages. As reported by Selkrig et al. (2020) in their spatiotemporal proteomic study, infected macrophages secreted large amounts of lysosomal hydrolases, including Cts C, Cts L, Cts D, Cts Z, and Cts A to the extracellular space, part of which were trafficked to the nucleus. Nuclear cathepsins were active and had a higher molecular weight compared to their lysosomal counterparts. Nuclear cathepsin activity was responsible for pyroptosis through the non-canonical inflammasome activation (Selkrig et al., 2020).

### *Mycoplasma sp.*

Mycoplasmas are the smallest self-replicating bacteria that completely lack a cell wall. They colonize mucosal surfaces of respiratory and urogenital tracts in humans and many different animal species. Mycoplasmas are mainly facultative intracellular organisms, however some of them are considered obligatory intracellular microorganisms (Nascimento et al., 2005). Among pathogenic mycoplasma species, those of greatest clinical importance for humans are: *M. pneumoniae*, associated mainly with atypical and community-acquired pneumonia (CAP); *M. genitalium*, responsible for acute and chronic urethritis in men; and *M. hominis* that causes inflammation of the urethra, cervix and vagina in women. *M. pneumoniae* and *M. genitalium* are also involved in pelvic inflammatory disease (Metwally et al., 2014).

*In vitro* and *in vivo* studies point to macrophages are as the major cells engaged in elimination of mycoplasma (Erb and Bredt, 1979; Schimmelpfeng et al., 1980; Hickman-Davis et al., 1997; Hickman-Davis et al., 1999; Lai et al., 2010). The macrophage-mediated killing of bacteria uses the MyD88-NF-κB pathway, as shown in a mouse model of *M. pneumoniae* infection (Lai et al., 2010) where bone marrow-derived macrophages (BMDMs) were able to phagocytose bacteria followed by formation of phagosomal compartment that eventually fused with lysosomes to form an acidified phagolysosome (Lai et al., 2010). It is therefore possible that cathepsin proteases may participate in elimination and control of mycoplasma infections. Cts L-deficient mice present significantly lower percentage of macrophages and higher mycoplasma burden in lungs and exhibit more severe pneumonia, compared to WT animals, following infection with *M. pulmonis*. Cts L probably has no direct toxic effect on mycoplasma, since it alters neither bacterial viability nor growth of *M. pulmonis* incubated with this protease *in vitro.* Instead, Cts L indirectly controls mycoplasma infection by promoting lymphangiogenesis and antibacterial cellular immune responses (Xu et al., 2013). Cts L can also promote mucosal immune response, which provides protection against mycoplasma pneumonia in piglets infected with *M. hyopneumoniae*. Enhancement of mucosal response by Cts L is mediated by stimulation of sIgA secretion through efficient Ii processing and antigen presentation. Treatment of animals with rCts L before challenge with *M. hyopneumoniae* resulted in milder clinical symptoms, little histopathological damage of lungs and lower mycoplasma burden accompanied by higher secretion of sIgA, higher percentages of CD4^+^ T cells and increased expression of MHC II molecules compared to the control group without rCts L exposure. Interestingly, Cts L levels were higher in DC than macrophages in most tissues of piglets infected with *M. hyopneumoniae* (Zhang et al., 2019).

## Obligate Intracellular Bacteria

### *Coxiella burnetii*

*C. burnetii*, a small Gram-negative bacterium, is the etiological agent of Q fever that infects a wide range of animals, from arthropods to humans. Q fever can cause acute or chronic infection, and the most frequent clinical sign of chronic Q fever is endocarditis (Eldin et al., 2017). This pathogen exhibits strong tropism to mononuclear phagocytes, such as monocytes and macrophages. It possesses a unique ability to replicate in a lysosome-like intracellular niche termed the *Coxiella*-containing vacuole (CCV) through activation of a Dot/Icm-type IVB secretion system. This secretion system allows the pathogen to translocate a large repertoire of effectors to the host cytosol, allowing the remodeling of host cell processes and the creation of a replicative niche while still maintaining the host cell homeostasis (Newton et al., 2020). *C. burnetii* undergoes phase variation with antigenic transition from a virulent phase I (characterized by the presence of full LPS) to an avirulent phase II (containing severely truncated LPS that lacks the O antigen and some core sugars) upon serial passages in cell cultures or embryonated eggs (Hotta et al., 2002; Howe et al., 2010).

Virulent and avirulent *C. burnetii* variants are phagocytosed by macrophages and are localized within early CCV containing endosome antigen 1 (EEA-1) and the small GTPase Rab5. The early CCV is then converted into a late CCV containing lysosome-associated membrane protein 1 (LAMP-1), LAMP-2, LAMP-3 (CD63), the mannose-6-phosphate receptor (M6PR), flotillin 1 and 2, autophagosome markers LC3 and the vacuolar type H^+^ ATPase responsible for the moderately acidic pH (pH ∼5) of the compartment (Ghigo et al., 2002; Voth and Heinzen, 2007; Howe et al., 2010). Virulent *C. burnetii* blocks maturation of CCV at late endosomal stage through inhibition of Cts D and small GTPase Rab7 recruitment, thus avoiding degradation in the phagolysosome and increasing pathogen survival. Conversely, CCV hiding avirulent *C. burnetii* recruits Rab7 and matures into bactericidal phagolysosomal compartment that contains active lysosomal enzymes, including Cts D. This trafficking behavior correlates with avirulent *C. burnetii* elimination by THP-1 macrophage-like cells (Ghigo et al., 2002). However, these prior findings are contradicted by Howe et al. (2010) who reported that CCV harboring virulent or avirulent variants of *C. burnetii* mature similarly through the endolysosomal route and reach the phagolysosome stage containing proteolytically active cathepsins. Another study supports the notion that lysosomal hydrolases, including Cts D, are not required for *C. burnetii* growth or viability in CCV (Miller et al., 2019). Specific antibodies found in chronic Q fever patients likely favor *C. burnetii* replication in human MDM, because opsonization of bacteria with these antibodies prevented the phagosome conversion. The large CCV containing *C. burnetii* found in such cases were devoid of a specific phagolysosome marker Cts D (Desnues et al., 2009).

*C. burnetii* down-regulates Cts B activity, but does not significantly affect LAMP-1 in murine alveolar macrophages (MH-S) or HeLa cells, suggesting that cells may contain less proteolytically-active lysosomes. Reduction of lysosomal content in *C. burnetii*-infected cells was independent of autophagy process. Additionally, HeLa cells overexpressing the transcription factor EB (TFEB), which coordinates expression of lysosomal genes for lysosomal proteases and hydrolases, led to increased Cts B activity, indicating an increase in the number of proteolytically-active lysosomes. TFEB-induced lysosome biogenesis significantly reduced *C*. *burnetii* CCV formation and intracellular growth due to acidification and increased protease activity (Samanta et al., 2019). These data underscore the importance of Cts B in the control of *C*. *burnetii* intracellularly and present a mechanism where a bacterial intracellular niche is created by regulating the acidity of the vacuoles-containing bacteria and blocking endosomal maturation.

### *Chlamydia spp.*

Gram-negative *C. trachomatis* is a leading cause of sexually transmitted infections in humans worldwide. In women, it can cause cervicitis, and in both men and women the infection can lead to urethritis and proctitis. Mice inoculated with the murine pathogen *C. muridarum* serve as a model of *C. trachomatis* infections in women (Conrad et al., 2016)*. Chlamydia* exists in two morphologically distinct forms: elementary body (EB), which is infectious and extracellular, and reticulate body (RB), which is intracellular and divides within a specialized intracellular vacuole forming a microcolony, termed an ‘inclusion’ (Kosma, 1999). During experimental genital infection with *Chlamydia*, there is recruitment of monocytes and macrophages to the genital tract which phagocytose and eliminate the pathogen intracellularly, limiting the development of disease. However, when intracellular killing is not effective, especially in M2 macrophages, bacteria can be easily disseminated to the lymphatic system and further replicate in the draining lymph nodes (Lausen et al., 2018; Tietzel et al., 2019).

*C. trachomatis* may survive within macrophages by inhibiting fusion between *Chlamydia* vacuoles and lysosomes (Coutinho-Silva et al., 2003), thus limiting access to lysosomal enzymes, including cathepsins. Cts B activity was increased in murine RAW 264.7 macrophages infected with a moderate dose of *C. muridarum*, and treatment of infected cells with Ca-074Me, a selective Cts B inhibitor, resulted in increased production of chlamydial inclusion forming units (IFU). Cts B activity depended on ROS production, because incubation of infected macrophages with *N*,*N*′-dimethylthiourea (DMTU), a potent scavenger *of* hydroxyl radicals, significantly reduced activity of this protease. In macrophages infected with *C. muridarum*, Cts B and ROS were involved in activation of inducible nitric oxide synthase (iNOS) that correlated with and was responsible for chlamydial clearance (Rajaram and Nelson, 2015). Furthermore, the accumulation of active lysosomal protease Cts D within bacteria inclusions has been demonstrated in different types of *C. muridanum*-infected cells, including THP-1 macrophages, after treatment with penicillin G (pG). Accumulation of Cts D clearly preceded the decrease in transcription of pre-16S rRNA, suggesting the involvement of this protease in bacterial death. Activation of Cts D in pG treatment was independent of the activation of lysosomal H+v-ATPase; therefore, the mechanism of lysosomes fusion with the bacterial vacuoles in pG-treated infected cells remains to be elucidated (Dumoux et al., 2013). Cts D and Cts S are engaged in MHC I–mediated cross-presentation pathway of chlamydial antigens in DCs required for efficient stimulation of bacteria-specific CD8^+^ T cells (Fiegl et al., 2013).

### *Rickettsia spp.*

Gram-negative rickettsiae are important causative agents of various ailments within the group of arthropod-borne diseases. *Rickettsia* are classified into four distinct groups based on their genome sequences, including the typhus group (TG with *Rickettsia typhi* and *Rickettsia prowazekii*), the spotted fever group (SFG), the ancestral group (AG), and the transitional group (TRG) (Snowden and Bhimji, 2018). Macrophages may play an important role in the pathogenesis of rickettsial diseases, because various bacteria species show some ability to proliferate within these cells depending on their virulence (Curto et al., 2016; Curto et al., 2019a). The dichotomy regarding *Rickettsia* ability to survive within macrophages has been extensively documented in SPG members that cause human tick-borne diseases with varying severity (Curto et al., 2016; Curto et al., 2019a; Curto et al., 2019b). A pathogenic *R. conorii* is responsible for Mediterranean spotted fever (MSF) associated with high morbidity and mortality, whereas non-pathogenic *R. montanensis* causes infections with mild or no systemic symptoms in humans (Rovery et al., 2008; McQuiston et al., 2012). In THP-1 macrophages, *R. conorii* cells display normal morphology and do not co-localize with lysosomal markers, Cts D and LAMP-2, suggesting that the bacteria do not enter the phagolysosomal compartment and/or can escape from the phagosome to the cytosol. Therefore, they can efficiently proliferate within THP-1 cells. In contrast, *R. montanensis* co-localizes with Cts D and LAMP-2 and is rapidly destroyed, thus disrupting its ability to survive and proliferate in THP-1 macrophages (Curto et al., 2016). Transcriptomic analysis using RNA-seq revealed down-regulation of *Ctsd* and *Ctsz* mRNA expression in THP-1 macrophages infected either with *R. conorii* or *R. montanensis* (Curto et al., 2019a), whereas quantitative proteomic studies using a SWATH*-*MS strategy indicated decreased Cts G level only in macrophages infected with *R. conorii (* Curto et al., 2019b *).* Overall, pathogenic and non-pathogenic SFG *Rickettsia* trigger differential transcriptomic and proteomic signatures in THP-1 cells, what may partially explain different intracellular fates of these pathogens within macrophages (Curto et al., 2019a; Curto et al., 2019b). It should be also added that pathogenic SFG rickettsiae may possibly increase their intracellular survival within macrophages by avoidance of digestion by lysosomal enzymes, including cathepsins. Therefore, the role of these proteases in the host response to *Rickettsia* infection should be elucidated in future studies.

## Conclusion

Cathepsins are undoubtedly important proteases that participate in killing various bacterial pathogens both directly or indirectly. Experimental evidence indicates that survival of many bacterial species within macrophages is achieved by pathogen interference with intracellular trafficking events, leading to disruptions of fusion events between the phagosome and lysosomes, modification of the intraphagosomal environment or escape from the phagosome/phagolysosome into the host cytosol. In doing so, pathogens escape toxic bactericidal lysosomal compounds, including cathepsins, therefore, they can survive and even replicate within specialized engulfing cells, such as macrophages. Further, certain intracellular pathogens are able to modify and exclude cathepsins from bacteria-containing vacuoles, allowing them to adapt to this hostile endolysosomal system as a niche for efficient growth. Cathepsins regulate many innate functions of macrophages including those that support adaptive immune responses that ultimately control of bacterial infections. Consequently, bacterial pathogens manipulate expression, activity and bioavailibility of cathepsins, thus compromising their ability to kill bacteria, and ultimately leading to disease exacerbation. However, the exact mechanisms engaged by bacteria remain unclear in many cases and further investigation are warranted to better define their role in human diseases and to identify new therapeutic targets or vaccines. Because cathepsins are enzymes they can directly digest pathogens and thus contribute to derivation of antigenic epitopes required for the generation of adaptive immunity. However, many studies summarized in this review were performed using *in vitro*-generated murine macrophages or leukemia-derived monocytic cell lines, which do not accurately reflect physiological conditions. Therefore, the role of cathepsins in regulation of mechanisms of the antimicrobial immune response and pathogenesis of bacterial diseases requires further *in vivo* studies in animal models and humans.

## Author Contributions

LS-D conceptualized the article. LS-D and MB-N wrote the draft of the manuscript. All authors contributed to the article and approved the submitted version.

## Conflict of Interest

The authors declare that the research was conducted in the absence of any commercial or financial relationships that could be construed as a potential conflict of interest.

## Acknowledgments

We sincerely apologize to those authors whose original publications were not cited in this review due to space limitation. We would like to thank Ada, Anastazja, Konstanty Dabrowscy, and Tobiasz Turbak for their invaluable assistance during preparation of this manuscript.

## References

[B1] Abd-Elrahman I. Meir K. Kosuge H. Ben-Nun Y. Weiss Sadan T. Rubinstein C. (2016). Characterizing cathepsin activity and macrophage subtypes in excised human carotid plaques. Stroke 47, 1101–1108. 10.1161/STROKEAHA.115.011573 26941255

[B2] Alam S. Liu G. Liu S. Liu Y. Zhang Y. Yang X. (2019). Up-regulated cathepsin C induces macrophage M1 polarization through FAK-triggered p38 MAPK/NF-κB pathway. Exp. Cell. Res. 382 (2), 111472. 10.1016/j.yexcr.2019.06.017 31229505

[B3] Alford C. E. Amaral E. Campbell P. A. (1990). Listericidal activity of human neutrophil cathepsin G. J. Gen. Microbiol. 136 (6), 997–100. 10.1099/00221287-136-6-997 2117044

[B4] Alpuche-Aranda C. M. Racoosin E. L. Swanson J. A. Miller S. II (1994). Salmonella stimulate macrophage macropinocytosis and persist within spacious phagosomes. J. Exp. Med. 179 (2), 601–608. 10.1084/jem.179.2.601 8294870 PMC2191354

[B5] Amaral E. P. Riteau N. Moayeri M. Maier N. Mayer-Barber K. D. Pereira R. M. (2018). Lysosomal cathepsin release is required for NLRP3-inflammasome activation by *Mycobacterium tuberculosis* in infected macrophages. Front. Immunol. 9:1427:1427. 10.3389/fimmu.2018.01427 29977244 PMC6021483

[B6] Arpaia N. Godec J. Lau L. Sivick K. E. McLaughlin L. M. Jones M. B. (2011). TLR signaling Is required for *Salmonella typhimurium* virulence. Cell 144 (5), 675–688. 10.1016/j.cell.2011.01.031 21376231 PMC3063366

[B7] Asare R. Kwaik Y. A. (2011). Exploitation of host cell biology and evasion of immunity by *Francisella tularensis* . Front. Microbiol. 1:145:145. 10.3389/fmicb.2010.00145 21687747 PMC3109322

[B8] Ashida H. Kim M. Sasakawa C. (2014). Manipulation of the host cell death pathway by Shigella. Cell. Microbiol. 16 (12), 1757–1766. 10.1111/cmi.12367 25264025

[B9] Baena A. Porcelli S. A. (2009). Evasion and subversion of antigen presentation by Mycobacterium tuberculosis. Tissue Antigens 74 (3), 189–204. 10.1111/j.1399-0039.2009.01301.x 19563525 PMC2753606

[B10] Bakaletz L. O. DeMaria T. F. Lim D. J. (1987). Phagocytosis and killing of bacteria by middle ear macrophages. Arch. Otolaryngol. Head Neck Surg. 113 (2), 138–144. 10.1001/archotol.1987.01860020030007 3492213

[B11] Barrett A. J. Rawlings N. D. Woessner J. F. (2004). Handbook of proteolytic enzymes (New York, USA: Academic Press).

[B12] Bastaert F. Kheir S. Saint-Criq V. Villeret B. Dang P. M. C. El-Benna J. (2018). *Pseudomonas aeruginosa* LasB subverts alveolar macrophage activity by interfering with bacterial killing through downregulation of innate immune defense, reactive oxygen species generation, and complement activation. Front. Immunol. 9:1675. 10.3389/fimmu.2018.01675 30083156 PMC6064941

[B13] Beers C. Honey K. Fink S. Forbush K. Rudensky A. (2003). Differential regulation of cathepsin S and cathepsin L in interferon γ–treated macrophages. J. Exp. Med. 197 (2), 169–179. 10.1084/jem.20020978 12538657 PMC2193812

[B14] Belaia I. A. Khasman E. L. Popova L. II (1984). Cathepsin D activity in splenocytes in relation to *Shigella* virulence in an experiment. Zh. Mikrobiol. Epidemiol. Immunobiol. 11, 72–76. 6395592

[B15] Bellaire B. H. Roop 2nd R. M. Cardelli J. A. (2005). Opsonized virulent *Brucella abortus* replicates within nonacidic, endoplasmic reticulum-negative, LAMP-1-positive phagosomes in human monocytes. Infect. Immun. 73 (6), 3702–3713. 10.1128/IAI.73.6.3702-3713.2005 15908400 PMC1111828

[B16] Bennett K. Levine T. Ellis J. S. Peanasky R. J. Samloff I. M. Kay J. (1992). Antigen processing for presentation by class II major histocompatibility complex requires cleavage by cathepsin E. Eur. J. Immunol. 22, 1519–1524. 10.1002/eji.1830220626 1601038

[B17] Benoit M. Desnues B. Mege J. L. (2008). Macrophage polarization in bacterial infections. J. Immunol. 181 (6), 3733–3739. 10.4049/jimmunol.181.6.3733 18768823

[B18] Bever C. T. Jr. Morgan K. D. Whitaker J. N. (1989). Cathepsin D activity in human peripheral blood mononuclear leukocytes. Inflammation 13 (3), 309–316. 10.1007/BF00914397 2787785

[B19] Bewley M. A. Marriott H. M. Tulone C. Francis S. E. Mitchell T. J. Read R. C. (2011a). A cardinal role for cathepsin D in co-ordinating the host-mediated apoptosis of macrophages and killing of pneumococci. PloS Pathog. 7 (1), e1001262. 10.1371/journal.ppat.1001262 21298030 PMC3029254

[B20] Bewley M. A. Pham T. K. Marriott H. M. Noirel J. Chu H. P. Ow S. Y. (2011b). Proteomic evaluation and validation of cathepsin D regulated proteins in macrophages exposed to *Streptococcus pneumonia* . Mol. Cell. Proteomics 10 (6):M111.008193. 10.1074/mcp.M111.008193 PMC3108842 21474794

[B21] Bhagirath A. Y. Li Y. Somayajula D. Dadashi M. Badr S. Duan K. (2016). Cystic fibrosis lung environment and *Pseudomonas aeruginosa* infection. BMC Pulm. Med. 16, 174. 10.1186/s12890-016-0339-5 27919253 PMC5139081

[B22] Bhat K. H. Mukhopadhyay S. (2015). Macrophage takeover and the host-bacilli interplay during tuberculosis. Future Microbiol. 10 (5), 853–872. 10.2217/fmb.15.11 26000654

[B23] Bradford M. K. Elkins K. L. (2020). Immune lymphocytes halt replication of *Francisella tularensis* LVS within the cytoplasm of infected macrophages. Sci. Rep. 10 (1), 12023. 10.1038/s41598-020-68798-2 32694562 PMC7374111

[B24] Brann K. R. Fullerton M. S. Onyilagha F. II Prince A. A. Kurten R. C. Rom J. S. (2019). Infection of primary human alveolar macrophages alters *Staphylococcus aureus* toxin production and activity. Infect. Immun. 87 (7), e00167–e00119. 10.1128/IAI.00167-19 31010814 PMC6589068

[B25] Brown R. Nath S. Lora A. Samaha G. Elgamal Z. Kaiser R. (2020). Cathepsin S: investigating an old player in lung disease pathogenesis, comorbidities, and potential therapeutics. Respir. Res. 21, 111. 10.1186/s12931-020-01381-5 32398133 PMC7216426

[B26] Bruscia E. M. Bonfield T.,. L. (2016). Cystic fibrosis lung immunity: the role of the macrophage. J. Innate Immun. 8 (6), 550–563. 10.1159/000446825 27336915 PMC5089923

[B27] Bühling F. Reisenauer A. Gerber A. Krüger S. Weber E. Brömme D. (2001). Cathepsin K–a marker of macrophage differentiation? J. Pathol. 195 (3), 375–382. 10.1002/path.959 11673837

[B28] Burster T. Macmillan H. Hou T. Boehm B. O. Mellins E. D. (2010). Cathepsin G: roles in antigen presentation and beyond. Mol. Immunol. 47, 658–665. 10.1016/j.molimm.2009.10.003 19910052 PMC4159238

[B29] Carrasco-Marín E. Madrazo-Toca F. de los Toyos J. R. Cacho-Alonso E. Tobes R. Pareja E. (2009). The innate immunity role of cathepsin-D is linked to Trp-491 and Trp-492 residues of listeriolysin O. Mol. Microbiol. 72 (3), 668–682. 10.1111/j.1365-2958.2009.06673.x 19389128

[B30] Celli J. de Chastellier C. Franchini D. M. Pizarro-Cerda J. Moreno E. Gorvel J. P. (2003). *Brucella* evades macrophage killing *via* VirB-dependent sustained interactions with the endoplasmic reticulum. J. Exp. Med. 198 (4), 545–556. 10.1084/jem.20030088 12925673 PMC2194179

[B31] Chain B. M. Free P. Medd P. Swetman C. Tabor A. B. Terrazzini N. (2005). The expression and function of cathepsin E in dendritic cells. J. Immunol. 174 (4), 1791–1800. 10.4049/jimmunol.174.4.1791 15699105

[B32] Château A. Seifert S. (2016). *Neisseria gonorrhoeae* survives within and modulates apoptosis and inflammatory cytokine production of human macrophages. Cell. Microbiol. 18 (4), 546–560. 10.1111/cmi.12529 26426083 PMC5240846

[B33] Chen D. Xie J. Fiskesund R. Dong W. Liang X. Lv J. (2018). Chloroquine modulates antitumor immune response by resetting tumor-associated macrophages toward M1 phenotype. Nat. Commun. 9 (1), 873. 10.1038/s41467-018-03225-9 29491374 PMC5830447

[B34] Chwieralski C. E. Welte T. Bühling F. (2006). Cathepsin-regulated apoptosis. Apoptosis 11 (2), 143–149. 10.1007/s10495-006-3486-y 16502253

[B35] Clemens D. L. Lee B. Y. Horwitz M. A. (2004). Virulent and avirulent strains of *Francisella tularensis* prevent acidification and maturation of their phagosomes and escape into the cytoplasm in human macrophages. Infect. Immun. 72 (6), 3204–3217. 10.1128/IAI.72.6.3204-3217.2004 15155622 PMC415696

[B36] Conrad T. A. Gong S. Yang Z. Matulich P. Keck J. Beltrami N. (2016). The chromosome-encoded hypothetical protein TC0668 is an upper genital tract pathogenicity factor of *Chlamydia muridarum* . Infect. Immun. 84 (2), 467–479. 10.1128/IAI.01171-15 26597987 PMC4730586

[B37] Conus S. Simon H. U. (2010). Cathepsins and their involvement in immune responses. Swiss Med. Wkly. 140, w13042. 10.4414/smw.2010.13042 20648403

[B38] Coria L. M. Ibañez A. E. Tkach M. Sabbione F. Bruno L. Carabajal M. V. (2016). A *Brucella* spp. protease inhibitor limits antigen lysosomal proteolysis, increases cross-presentation, and enhances CD8+ T cell responses. J. Immunol. 196 (10), 4014–4029. 10.4049/jimmunol.1501188 27084100

[B39] Coutinho-Silva R. Stahl L. Raymond M. N. Jungas T. Verbeke R. Burnstock G. (2003). Inhibition of chlamydial infectious activity due to P2X7R-dependent phospholipase D activation. Immunity 19 (3), 403–412. 10.1016/s1074-7613(03)00235-8 14499115

[B40] Creasy B. M. McCoy K. L. (2011). Cytokines regulate cysteine cathepsins during TLR responses. Cell. Immunol. 267 (1), 56–66. 10.1016/j.cellimm.2010.11.004 21145045 PMC3032264

[B41] Curto P. Simões I. Riley S. P. Martinez J. J. (2016). Differences in intracellular fate of two spotted fever group *Rickettsia* in macrophage-like cells. Front. Cell. Infect. Microbiol. 6:80:80. 10.3389/fcimb.2016.00080 27525249 PMC4965480

[B42] Curto P. Riley S. P. Simões I. Martinez J. J. (2019a). Macrophages infected by a pathogen and a non-pathogen spotted fever group *Rickettsia* reveal differential reprogramming signatures early in infection. Front. Cell. Infect. Microbiol. 9:97:97. 10.3389/fcimb.2019.00097 31024862 PMC6467950

[B43] Curto P. Santa C. Allen P. Manadas B. Simões I. Martinez J. J. (2019b). A pathogen and a non-pathogen spotted fever group *Rickettsia* trigger differential proteome signatures in macrophages. Front. Cell. Infect. Microbiol. 9:43:43. 10.3389/fcimb.2019.00043 30895174 PMC6414445

[B44] Dalpke A. H. Helm M. (2012). RNA mediated toll-like receptor stimulation in health and disease. RNA Biol. 9 (6), 828–842. 10.4161/rna.20206 22617878 PMC3495747

[B45] Danelishvili L. Everman J. L. McNamara M. J. Bermudez L. E. (2012). Inhibition of the plasma-membrane-associated serine protease cathepsin G by *Mycobacterium tuberculosis* Rv3364c suppresses caspase-1 and pyroptosis in macrophages. Front. Microbiol. 2:281:281. 10.3389/fmicb.2011.00281 22275911 PMC3257866

[B46] del Cerro-Vadillo E. Madrazo-Toca F. Carrasco-Marín E. Fernandez-Prieto L. Beck C. Leyva-Cobián F. L. (2006). Cutting edge: a novel nonoxidative phagosomal mechanism exerted by cathepsin-D controls *Listeria monocytogenes* intracellular growth. J. Immunol. 176 (3), 1321–1325. 10.4049/jimmunol.176.3.1321 16424157

[B47] Deng Q. Wang Y. Zhang Y. Li M. Li D. Huang X. (2016). *Pseudomonas aeruginosa* triggers macrophage autophagy to escape intracellular killing by activation of the NLRP3 inflammasome. Infect. Immun. 84 (1), 56–66. 10.1128/IAI.00945-15 26467446 PMC4694000

[B48] Desnues B. Imbert G. Raoult D. Mege J. L. Ghigo E. (2009). Role of specific antibodies in *Coxiella burnetii* infection of macrophages. Clin. Microbiol. Infect. 15 (s2), 161–162. 10.1111/j.1469-0691.2008.02208.x 19281459

[B49] Dockrell D. H. Lee M. Lynch D. H. Read R. C. (2001). Immune-mediated phagocytosis and killing of Streptococcus pneumoniae are associated with direct and bystander macrophage apoptosis. J. Infect. Dis. 184 (6), 713–722. 10.1086/323084 11517432

[B50] Dumoux M. Le Gall S. M. Habbeddine M. Delarbre C. Hayward R. D. Kanellopoulos-Langevin C. (2013). Penicillin kills *Chlamydia* following the fusion of bacteria with lysosomes and prevents genital inflammatory lesions in *C. muridarum*-infected mice. PloS One 8 (12), e83511. 10.1371/journal.pone.0083511 24376710 PMC3871543

[B51] Duncan J. A. Gao X. Huang M. T. H. O’Connor B. P. Thomas C. E. Willingham S. B. (2009). *Neisseria gonorrhoeae* activates the proteinase cathepsin B to mediate the signaling activities of the NLRP3 and ASC-containing inflammasome. J. Immunol. 182 (10), 6460–6469. 10.4049/jimmunol.0802696 19414800 PMC2722440

[B52] Eldin C. Mélenotte C. Mediannikov O. Ghigo E. Million M. Edouard S. (2017). From Q fever to *Coxiella burnetii* infection: a paradigm change. Clin. Microbiol. Rev. 30 (1), 115–190. 10.1128/CMR.00045-16 27856520 PMC5217791

[B53] Erb P. Bredt W. (1979). Interaction of *Mycoplasma pneumoniae* with alveolar macrophages: viability of adherent and ingested mycoplasmas. Infect. Immun. 25 (1), 11–15. 10.1128/IAI.25.1.11-15.1979 113340 PMC414413

[B54] Ewald S. E. Engel A. Lee J. Wang M. Bogyo M. Barton G. M. (2011). Nucleic acid recognition by Toll-like receptors is coupled to stepwise processing by cathepsins and asparagine endopeptidase. J. Exp. Med. 208 (4), 643–651. 10.1084/jem.20100682 21402738 PMC3135342

[B55] Farber J. M. Peterkin P. II (1991). *Listeria monocytogenes*, a food-borne pathogen. Microbiol. Rev. 55 (3), 476–511. 10.1128/MMBR.55.3.476-511.1991 1943998 PMC372831

[B56] Fiegl D. Kägebein D. Liebler-Tenorio E. M. Weisser T. Sens M. Gutjahr M. (2013). Amphisomal route of MHC class I cross-presentation in bacteria-infected dendritic cells. J. Immunol. 190 (6), 2791–2806. 10.4049/jimmunol.1202741 23418629

[B57] Fu H. Ma Y. Yang M. Zhang C. Huang H. Xia Y. (2016). Persisting and increasing neutrophil infiltration associates with gastric carcinogenesis and E-cadherin downregulation. Sci. Rep. 6:29762. 10.1038/srep29762 27412620 PMC4944193

[B58] Fu Q. Yuan J. Wang L. Ran H. Li F. Liu F. (2020). Proteomic analysis of murine macrophages mitochondria and lysosomes reveal Cathepsin D as a potential broad-spectrum antimicrobial protein. J. Proteomics 223:103821. 10.1016/j.jprot.2020.103821 32417293

[B59] Ghigo E. Capo C. Tung C. H. Raoult D. Gorvel J. P. Mege J. L. (2002). *Coxiella burnetii* survival in THP-1 monocytes involves the impairment of phagosome maturation: IFN-gamma mediates its restoration and bacterial killing. J. Immunol. 169 (8), 4488–4495. 10.4049/jimmunol.169.8.4488 12370385

[B60] Gogoi M. Shreenivas M. M. Chakravortty D. (2019). Hoodwinking the big-eater to prosper: the salmonella-macrophage paradigm. J. Innate Immun. 11 (3), 289–299. 10.1159/000490953 30041182 PMC6738159

[B61] Gómez L. A. Alvarez F. II Molina R. E. Soto-Shara R. Daza-Castro C. Flores M. R. (2020). A zinc-dependent metalloproteinase of *Brucella abortus* is required in the intracellular adaptation of macrophages. Front. Microbiol. 11:1586. 10.3389/fmicb.2020.01586 32765455 PMC7379133

[B62] Grzonka Z. Jankowska E. Kasprzykowski F. Kasprzykowska R. Lankiewicz L. Wiczk W. (2001). Structural studies of cysteine proteases and their inhibitors. Acta Biochim. Pol. 48 (1), 1–20. 10.18388/abp.2001_5108 11440158

[B63] Guicciardi M. E. Leist M. Gores G. J. (2004). Lysosomes in cell death. Oncogene 23, 2881–2890. 10.1038/sj.onc.1207512 15077151

[B64] Guinn K. M. Rubin E. J. (2017). Tuberculosis: Just the FAQs. mBio 8 (6), e01910–e01917. 10.1128/mBio.01910-17 29259086 PMC5736911

[B65] Gutierrez M. G. Mishra B. B. Jordao L. Elliott E. Anes E. Griffiths G. (2008). NF-kappa B activation controls phagolysosome fusion-mediated killing of mycobacteria by macrophages. J. Immunol. 181 (4), 2651–2663. 10.4049/jimmunol.181.4.2651 18684956

[B66] Ha S. D. Martins A. Khazaie K. Han J. Chan B. M. C. Kim S. O. (2008). Cathepsin B is involved in the trafficking of TNF-alpha-containing vesicles to the plasma membrane in macrophages. J. Immunol. 181 (1), 690–697. 10.4049/jimmunol.181.1.690 18566436

[B67] Hahn I. Klaus I. Janze A. K. Steinwede K. Ding N. Bohling J. (2011). Cathepsin G and neutrophil elastase play critical and nonredundant roles in lung-protective immunity against *Streptococcus pneumoniae* in mice. Infect. Immun. 79 (12), 4893–4901. 10.1128/IAI.05593-11 21911460 PMC3232647

[B68] Hall J. D. Woolard M. D. Gunn B. M. Craven R. R. Taft-Benz S. Frelinger J. A. (2008). Infected-host-cell repertoire and cellular response in the lung following inhalation of Francisella tularensis Schu S4, LVS, or U112. Infect. Immun. 76 (12), 5843–5852. 10.1128/IAI.01176-08 18852251 PMC2583552

[B69] Hang H. C. Loureiro J. Spooner E. van der Velden A. W. M. Kim Y. M. Pollington A. M. (2006). Mechanism-based probe for the analysis of cathepsin cysteine proteases in living cells. ACS Chem. Biol. 1 (11), 713–723. 10.1021/cb600431a 17184136

[B70] Hannaford J. Guo H. Chen X. (2012). Involvement of cathepsins B and L in inflammation and cholesterol trafficking protein NPC2 secretion in macrophages. Obesity 21 (8), 1586–1595. 10.1002/oby.20136 PMC6445554 23666609

[B71] Herdendorf T. J. Stapels D. A. C. Rooijakkers S. H. M. Geisbrecht B. V. (2020). Local structural plasticity of the *Staphylococcus aureus* evasion protein EapH1 enables engagement with multiple neutrophil serine proteases. J. Biol. Chem. 295 (22), 7753–7762. 10.1074/jbc.RA120.013601 32303641 PMC7261791

[B72] Herías M. V. Biessen E. A. L. Beckers C. Delsing D. Liao M. Daemen M. J. (2015). Leukocyte cathepsin c deficiency attenuates atherosclerotic lesion progression by selective tuning of innate and adaptive immune responses. Arterioscler. Thromb. Vasc. Biol. 35 (1), 79–86. 10.1161/ATVBAHA.114.304292 25395616 PMC4270842

[B73] Hickman-Davis J. M. Michalek S. M. Gibbs-Erwin J. Lindsey J. R. (1997). Depletion of alveolar macropahges exacerbates respiratory mycoplasmosis in mycoplasma-resistant C57BL mice but not mycoplasma-susceptible C3H mice. Infect. Immun. 65 (6), 2278–2282. 10.1128/IAI.65.6.2278-2282.1997 9169764 PMC175316

[B74] Hickman-Davis J. Gibbs-Erwin J. Lindsey J. R. Matalon S. (1999). Surfactant protein A mediates mycoplasmacidal activity of alveolar macrophages by production of peroxynitrite. Proc. Natl. Acad. Sci. U.S.A. 96 (9), 4953–4958. 10.1073/pnas.96.9.4953 10220400 PMC21798

[B75] Hilgenberg E. Shen P. Dang V. D. Ries S. Sakwa I. Fillatreau S. (2014). Interleukin-10-producing B cells and the regulation of immunity. Curr. Top. Microbiol. Immunol. 380, 69–92. 10.1007/978-3-662-43492-5_4 25004814

[B76] Hirayama D. Iida T. Nakase H. (2018). The phagocytic function of macrophage-enforcing innate immunity and tissue homeostasis. Int. J. Mol. Sci. 19 (1):92. 10.3390/ijms19010092 PMC5796042 29286292

[B77] Hop H. T. Reyes A. W. B. Huy T. X. N. Arayan L. T. Min W. Lee H. J. (2018). Interleukin 10 suppresses lysosome-mediated killing of *Brucella abortus* in cultured macrophages. J. Biol. Chem. 293 (9), 3134–3144. 10.1074/jbc.M117.805556 29301939 PMC5836127

[B78] Hotta A. Kawamura M. To H. Andoh M. Yamaguchi T. Fukushi H. (2002). Phase variation analysis of *Coxiella burnetii* during serial passage in cell culture by use of monoclonal antibodies. Infect. Immun. 70 (8), 4747–4749. 10.1128/IAI.70.8.4747-4749.2002 12117996 PMC128212

[B79] Howe D. Shannon J. G. Winfree S. Dorward D. W. Heinzen R. A. (2010). *Coxiella burnetii* phase I and II variants replicate with similar kinetics in degradative phagolysosome-like compartments of human macrophages. Infect. Immun. 78 (8), 3465–3474. 10.1128/IAI.00406-10 20515926 PMC2916283

[B80] Hsieh C. S. deRoos P. Honey K. Beers C. Rudensky A. Y. (2002). A role for cathepsin L and cathepsin S in peptide generation for MHC class II presentation. J. Immunol. 168 (6), 2618–2625. 10.4049/jimmunol.168.6.2618 11884425

[B81] Jackman H. L. Tan F. L. Tamei H. Beurling-Harbury C. Li X. Y. Skidgel R. A. (1990). A peptidase in human platelets that deamidates tachykinins. Probable identity with the lysosomal “protective protein”. J. Biol. Chem. 265 (19), 11265–11272. 1694176

[B82] Jackman H. L. Tan F. Schraufnagel D. Dragovic T. Dezso B. Becker R. P. (1995). Plasma membrane-bound and lysosomal peptidases in human alveolar macrophages. Am. J. Respir. Cell. Mol. Biol. 13, 196–204. 10.1165/ajrcmb.13.2.7626287 7626287

[B83] Johnson M. B. Ball L. M. Daily K. P. Martin J. N. Columbus L. Criss A. K. (2014). Opa+ Neisseria gonorrhoeae exhibits reduced survival in human neutrophils *via* Src family kinase-mediated bacterial trafficking into mature phagolysosomes. Cell. Microbiol. 17 (5), 648–665. 10.1111/cmi.12389 25346239 PMC4402142

[B84] Jubrail J. Morris P. Bewley M. A. Stoneham S. Johnston S. A. Foster S. J. (2016). Inability to sustain intraphagolysosomal killing of *Staphylococcus aureus* predisposes to bacterial persistence in macrophages. Cell. Microbiol. 18 (1), 80–96. 10.1111/cmi.12485 26248337 PMC4778410

[B85] Kakehashi H. Nishioku T. Tsukuba T. Kadowaki T. Nakamura S. Yamamoto K. (2007). Differential regulation of the nature and functions of dendritic cells and macrophages by cathepsin E. J. Immunol. 179 (9), 5728–5737. 10.4049/jimmunol.179.9.5728 17947645

[B86] Kanno A. Tanimura N. Ishizaki M. Ohko K. Motoi Y. Onji M. (2015). Targeting cell surface TLR7 for therapeutic intervention in autoimmune diseases. Nat. Commun. 6, 6119. 10.1038/ncomms7119 25648980

[B87] Kawashima T. Kosaka A. Yan H. Guo Z. Uchiyama R. Fukui R. (2013). Double-stranded RNA of intestinal commensal but not pathogenic bacteria triggers production of protective interferon-β. Immunity 38 (6), 1187–1197. 10.1016/j.immuni.2013.02.024 23791646

[B88] Keim P. Johansson A. Wagner D. M. (2007). Molecular epidemiology, evolution, and ecology of *Francisella* . Ann. N. Y. Acad. Sci. 1105, 30–66. 10.1196/annals.1409.011 17435120

[B89] Kim S. Ock J. Kim A. K. Lee H. W. Cho J. Y. Kim D. R. (2007). Neurotoxicity of microglial cathepsin D revealed by secretome analysis. J. Neurochem. 103 (6), 2640–2650. 10.1111/j.1471-4159.2007.04995.x 17953665

[B90] Klevens R. M. Morrison M. A. Nadle J. Petit S. Gershman K. Ray S. (2007). Invasive methicillin-resistant *Staphylococcus aureus* infections in the United States. JAMA 298 (15), 1763–1771. 10.1001/jama.298.15.1763 17940231

[B91] Kos J. Jevnikar Z. Obermajer N. (2009). The role of cathepsin X in cell signaling. Cell Adh. Migr. 3, 164–166. 10.4161/cam.3.2.7403 19262176 PMC2679876

[B92] Kosma P. (1999). Chlamydial lipopolysaccharide. Biochim. Biophys. Acta 1455 (2-3), 387–402. 10.1016/s0925-4439(99)00061-7 10571027

[B93] Kotloff K. L. Nataro J. P. Blackwelder W. C. Nasrin D. Farag T. H. Panchalingamet S. (2013). Burden and aetiology of diarrhoeal disease in infants and young children in developing countries (the Global Enteric Multicenter Study, GEMS): a prospective, case-control study. Lancet 382 (9888), 209–222. 10.1016/S0140-6736(13)60844-2 23680352

[B94] Kotloff K. L. Riddle M. S. Platts-Mills J. A. Pavlinac P. Zaidi A. K. M. (2018). Shigellosis. Lancet 391 (10122), 801–812. 10.1016/S0140-6736(17)33296-8 29254859

[B95] Krueger S. Kalinski T. Hundertmark T. Wex T. Küster D. Peitz U. (2005). Up-regulation of cathepsin X in *Helicobacter pylori* gastritis and gastric cancer. J. Pathol. 207 (1), 32–42. 10.1002/path.1820 16025436

[B96] Krueger S. Kuester D. Bernhardt A. Wex T. Roessner A. (2009). Regulation of cathepsin X overexpression in *H. pylori*-infected gastric epithelial cells and macrophages. J. Pathol. 217 (4), 581–588. 10.1002/path.2485 19090485

[B97] Krueger S. Bernhardt A. Kalinski T. Baldensperger M. Zeh M. Teller A. (2013). Induction of premalignant host responses by cathepsin X/Z-deficiency in *Helicobacter pylori-*infected mice. PloS One 8 (7), e70242. 10.1371/journal.pone.0070242 23936173 PMC3728094

[B98] Lacoma A. Cano V. Moranta D. Regueiro V. Domínguez-Villanueva D. Laabei M. (2017). Investigating intracellular persistence of *Staphylococcus aureus* within a murine alveolar macrophage cell line. Virulence 8 (8), 1761–1775. 10.1080/21505594.2017.1361089 28762868 PMC5810471

[B99] Lah T. T. Hawley M. Rock K. L. Goldberg A. L. (1995). Gamma-interferon causes a selective induction of the lysosomal proteases, cathepsins B and L, in macrophages. FEBS Lett. 363 (1-2), 85–89. 10.1016/0014-5793(95)00287-j 7729559

[B100] Lai J. F. Zindl C. L. Duffy L. B. Atkinson T. P. Jung Y. W. van Rooijen N. (2010). Critical role of macrophages and their activation *via* MyD88-NFκB signaling in lung innate immunity to *Mycoplasma pneumoniae* . PloS One 5 (12), e14417. 10.1371/journal.pone.0014417 21203444 PMC3009709

[B101] Lauer P. Chow M. Y. Loessner M. J. Portnoy D. A. Calendar R. (2002). Construction, characterization, and use of two *Listeria monocytogenes* site-specific phage integration vectors. J. Bacteriol. 184 (15), 4177–4186. 10.1128/jb.184.15.4177-4186.2002 12107135 PMC135211

[B102] Lausen M. Christiansen G. Bouet Guldbæk Poulsen T. Birkelund S. (2018). Immunobiology of monocytes and macrophages during *Chlamydia trachomatis* infection. Microbes Infect. 21 (2), 73–84. 10.1016/j.micinf.2018.10.007 30528899

[B103] Lawrence C. P. Kadioglu A. Yang A. L. Coward W. R. Chow S. C. (2006). The cathepsin B inhibitor, z-FA-FMK, inhibits human T cell proliferation *in vitro* and modulates host response to pneumococcal infection *in vivo* . J. Immunol. 177 (6), 3827–3836. 10.4049/jimmunol.177.6.3827 16951345

[B104] Lewis M. S. Danelishvili L. Rose S. J. Bermudez L. E. (2019). MAV_4644 Interaction with the host cathepsin Z protects *Mycobacterium avium* subsp. *hominissuis* from rapid macrophage killing. Microorganisms 7 (5):144. 10.3390/microorganisms7050144 PMC6560410 31117286

[B105] Li R. Zhou R. Wang H. Li W. Pan M. Yao X. (2019). Gut microbiota-stimulated cathepsin K secretion mediates TLR4-dependent M2 macrophage polarization and promotes tumor metastasis in colorectal cancer. Cell Death Differ. 26, 2447–2463. 10.1038/s41418-019-0312-y 30850734 PMC6889446

[B106] Liang F. Seyrantepe V. Landry K. Ahmad R. Ahmad A. Stamatos N. M. (2006). Monocyte differentiation up-regulates the expression of the lysosomal sialidase, Neu1, and triggers its targeting to the plasma membrane *via* major histocompatibility complex class II-positive compartments. J. Biol. Chem. 281 (37), 27526–27538. 10.1074/jbc.M605633200 16835219

[B107] Li-Korotky H. S. Swarts J. D. Hebda P. A. Doyle W. J. (2004). Cathepsin gene expression profile in rat acute pneumococcal otitis media. Laryngoscope 114, 1032–1036. 10.1097/00005537-200406000-00014 15179208

[B108] Liu W. Yan M. Liu Y. McLeish K. R. Coleman W. G. Jr. Rodgers G. P. (2012). Olfactomedin 4 inhibits cathepsin C-mediated protease activities, thereby modulating neutrophil killing of *Staphylococcus aureus* and *Escherichia coli* in mice. J. Immunol. 189 (5), 2460–2467. 10.4049/jimmunol.1103179 22844115 PMC3424379

[B109] Liu Y. G. Teng Y. S. Cheng P. Kong H. Lv P. Y. Mao F. Y. (2018). Abrogation of cathepsin C by *Helicobacter pylori* impairs neutrophil activation to promote gastric infection. FASEB J. 33 (4), 5018–5033. 10.1096/fj.201802016RR 30596522

[B110] Liuzzo J. P. Petanceska S. S. Devi L. A. (1999). Neurotrophic factors regulate cathepsin S in macrophages and microglia: A role in the degradation of myelin basic protein and amyloid beta peptide. Mol. Med. 5 (5), 334–343. 10.1007/BF03402069 10390549 PMC2230424

[B111] Löser R. Pietzsch J. (2015). Cysteine cathepsins: their role in tumor progression and recent trends in the development of imaging probes. Front. Chem. 3:37:37. 10.3389/fchem.2015.00037 26157794 PMC4477214

[B112] Luedtke C. C. Andonian S. Igdoura S. Hermo L. (2000). Cathepsin A is expressed in a cell- and region-specific manner in the testis and epididymis and is not regulated by testicular or pituitary factors. J. Histochem. Cytochem. 48, 1131–1146. 10.1177/002215540004800810 10898806

[B113] Ma J. Li H. (2018). The role of gut microbiota in atherosclerosis and hypertension. Front. Pharmacol. 9:1082:1082. 10.3389/fphar.2018.01082 30319417 PMC6167910

[B114] Maekawa Y. Himeno K. Ishikawa H. Hisaeda H. Sakai T. Dainichi T. (1998). Switch of CD4+ T cell differentiation from Th2 to Th1 by treatment with cathepsin B inhibitor in experimental leishmaniasis. J. Immunol. 161 (5), 2120–2127. 9725203

[B115] Magill S. S. Edwards J. R. Bamberg W. Beldavs Z. G. Dumyati G. Kainer M. A. (2014a). Multistate point-prevalence survey of health care–associated infections. N. Engl. J. Med. 370 (13), 1198–1208. 10.1056/NEJMoa1306801 24670166 PMC4648343

[B116] Magill S. S. Edwards J. R. Fridkin S. K. (2014b). Emerging infections program healthcare-associated infections and antimicrobial use prevalence survey team survey of health care-associated infections. N. Engl. J. Med. 370, 2542–2543. 10.1038/s41598-020-68798-2 24963580

[B117] Mahmood D. F. D. Jguirim-Souissi I. Khadija E.-H. Blondeau N. Diderot V. Amrani S. (2011). Peroxisome proliferator-activated receptor gamma induces apoptosis and inhibits autophagy of human monocyte-derived macrophages *via* induction of cathepsin L: potential role in atherosclerosis. J. Biol. Chem. 286 (33), 28858–28866. 10.1074/jbc.M111.273292 21700710 PMC3190693

[B118] Man S. M. Kanneganti T. D. (2016). Regulation of lysosomal dynamics and autophagy by CTSB/cathepsin B. Autophagy 12 (12), 2504–2505. 10.1080/15548627.2016.1239679 27786577 PMC5173259

[B119] Mathur R. Oh H. Zhang D. Park S. G. Seo J. Koblansky A. (2012). A mouse model of *Salmonella typhi* infection. Cell 151 (3), 590–602. 10.1016/j.cell.2012.08.042 23101627 PMC3500584

[B120] Mayer-Barber K. D. Andrade B. B. Oland S. D. Amaral E. P. Barber D. L. Gonzales J. (2014). Host-directed therapy of tuberculosis based on interleukin-1 and type I interferon crosstalk. Nature 511 (7507), 99–103. 10.1038/nature1348 24990750 PMC4809146

[B121] McComb S. Shutinoski B. Thurston S. Cessford E. Kumar K. Sad S. (2014). Cathepsins limit macrophage necroptosis through cleavage of Rip1 kinase. J. Immunol. 192 (12), 5671–5678. 10.4049/jimmunol.1303380 24799565

[B122] McQuiston J. H. Zemtsova G. Perniciaro J. Hutson M. Singleton J. Nicholson W. L. (2012). Afebrile spotted fever group Rickettsia infection after a bite from a *Dermacentor variabilis* tick infected with Rickettsia montanensis. Vector Borne Zoonotic Dis. 12, 1059–1061. 10.1089/vbz.2012.1078 23153005 PMC4699432

[B123] McSorley S. J. (2014). Immunity to intestinal pathogens: lessons learned from *Salmonella* . Immunol. Rev. 260 (1), 168–182. 10.1111/imr.12184 24942689 PMC4066191

[B124] Metwally M. A. Yassin A. S. Essam T. M. Hamouda H. M. Amin M. A. (2014). Detection, characterization, and molecular typing of human Mycoplasma spp. from major hospitals in Cairo, Egypt. ScientificWorldJournal 2014:549858. 10.1155/2014/549858 25506614 PMC4258911

[B125] Miller H. E. Hoyt F. H. Heinzen R. A. (2019). Replication of *Coxiella burnetii* in a lysosome-like vacuole does not require lysosomal hydrolases. Infect. Immun. 87 (11), e00493–e00419. 10.1128/IAI.00493-19 31405956 PMC6803326

[B126] Mills S. D. Finlay B. B. (1998). Isolation and characterization of *Salmonella typhimurium* and *Yersinia pseudotuberculosis*-containing phagosomes from infected mouse macrophages: *Y. pseudotuberculosis* traffics to terminal lysosomes where they are degraded. Eur. J. Cell Biol. 77 (1), 35–47. 10.1016/S0171-9335(98)80100-3 9808287

[B127] Moldovan A. Fraunholz M. J. (2018). In or out: phagosomal escape of *Staphylococcus aureus* . Cell. Microbiol. 21 (3), e12997. 10.1111/cmi.12997 30576050

[B128] Mrschtik M. Ryan K. M. (2015). Lysosomal proteins in cell death and autophagy. FEBS J. 282 (10), 1858–1870. 10.1111/febs.13253 25735653

[B129] Mukhopadhyay S. Plüddemann A. Gordon S. (2009). Macrophage pattern recognition receptors in immunity, homeostasis and self tolerance. Adv. Exp. Med. Biol. 653, 1–14. 10.1007/978-1-4419-0901-5_1 19799108 PMC7123833

[B130] Müller S. Faulhaber A. Sieber C. Pfeifer D. Hochberg T. Gansz M. (2014). The endolysosomal cysteine cathepsins l and K are involved in macrophage-mediated clearance of *Staphylococcus aureus* and the concomitant cytokine induction. FASEB J. 28 (1), 162–175. 10.1096/fj.13-232272 24036885

[B131] Murakami Y. Fukui Y. R. Motoi Y. Kanno A. Shibata T. Tanimura N. (2014). Roles of the cleaved N-terminal TLR3 fragment and cell surface TLR3 in double-stranded RNA sensing. J. Immunol. 193 (10), 5208–5217. 10.4049/jimmunol.1400386 25305318

[B132] Nakken B. Varga T. Szatmari I. Szeles L. Gyongyosi A. Illarionov P. A. (2011). Peroxisome proliferator-activated receptor γ-regulated cathepsin D is required for lipid antigen presentation by dendritic cells. J. Immunol. 187 (1), 240–247. 10.4049/jimmunol.1002421 21632707

[B133] Nascimento E. R. Pereira V. L. A. Nascimento M. G. F. Barreto M. L. (2005). Avian mycoplasmosis update. Braz. J. Poult. Sci. 7 (1), 1–9. 10.1590/S1516-635X2005000100001

[B134] Naudin C. Joulin-Giet A. Couetdic G. Plésiat P. Szymanska A. Gorna E. (2011). Human cysteine cathepsins are not reliable markers of Infection by *Pseudomonas aeruginosa* in cystic fibrosis. PloS One 6 (9), e25577. 10.1371/journal.pone.0025577 21980493 PMC3182231

[B135] Nepal R. M. Mampe S. Shaffer B. Erickson A. H. Bryant P. (2006). Cathepsin L maturation and activity is impaired in macrophages harboring *M. avium* and *M. tuberculosis* . Int. Immunol. 18 (6), 931–939. 10.1093/intimm/dxl029 16636015

[B136] Newton P. Thomas D. R. Reed S. C. O. Lau N. Xu B. Ong S. Y. (2020). Lysosomal degradation products induce *Coxiella burnetii* virulence. PNAS 117 (12), 6801–6810. 10.1073/pnas.1921344117 32152125 PMC7104363

[B137] Nishioku T. Hashimoto K. Yamashita K. Liou S. Y. Kagamiishi Y. Maegawa H. (2002). Involvement of cathepsin E in exogenous antigen processing in primary cultured murine microglia. J. Biol. Chem. 277, 4816–4822. 10.1074/jbc.M108382200 11719510

[B138] Obermajer N. Premzl A. Zavasnik-Bergant T. Turk B. Kos J. (2006). Carboxypeptidase cathepsin X mediates beta2-integrin-dependent adhesion of differentiated U-937 cells. Exp. Cell Res. 312 (13), 2515–2527. 10.1016/j.yexcr.2006.04.019 16774752

[B139] Obermajer N. Repnik U. Jevnikar Z. Turk B. Kreft M. Kos J. (2008). Cysteine protease cathepsin X modulates immune response *via* activation of β_2_ integrins. Immunology 124 (1), 76–88. 10.1111/j.1365-2567.2007.02740.x 18194276 PMC2434384

[B140] Obermajer N. Magister Š. Kopitar A. N. Tepeš B. Ihan A. Kos J. (2009). Cathepsin X prevents an effective immune response against *Helicobacter pylori* infection. Eur. J. Cell Biol. 88 (8), 461–471. 10.1016/j.ejcb.2009.03.003 19446361

[B141] Oliveira S. C. Giambartolomei G. H. Cassataro J. (2011). Confronting the barriers to develop novel vaccines against brucellosis. Expert Rev. Vaccines. 10 (9), 1291–1305. 10.1586/erv.11.110 21919619

[B142] Onishi K. Li Y. Ishii K. Hisaeda H. Tang L. Duan X. (2004). Cathepsin L is crucial for a Th1-type immune response during Leishmania major infection. Microbes Infect. 6 (5), 468–474. 10.1016/j.micinf.2004.01.008 15109961

[B143] Ostrowska H. (1997). Cathepsin A-like activity is possibly the main acidic carboxypeptidase in human platelets. Platelets 8 (5), 355–360. 10.1080/09537109777221 16793668

[B144] Pappas G. Akritidis N. Bosilkovski M. Tsianos E. (2005). Brucellosis. N. Engl. J. Med. 352, 2325–2336. 10.1056/NEJMra050570 15930423

[B145] Pawar K. Sharbati J. Einspanier R. Sharbati S. (2016). *Mycobacterium bovis* BCG interferes with miR-3619-5p control of cathepsin S in the process of autophagy. Front. Cell. Infect. Microbiol. 6:27:27. 10.3389/fcimb.2016.00027 27014637 PMC4783571

[B146] Pechous R. D. McCarthy T. R. Zahrt T. C. (2009). Working toward the future: insights into *Francisella tularensis* pathogenesis and vaccine development. Microbiol. Mol. Biol. Rev. 73 (4), 684–711. 10.1128/MMBR.00028-09 19946137 PMC2786580

[B147] Pires D. Marques J. Pombo J. P. Carmo N. Bettencourt P. Neyrolles O. (2016). Role of cathepsins in *Mycobacterium tuberculosis* survival in human macrophages. Sci. Rep. 6:32247. 10.1038/srep32247 27572605 PMC5004184

[B148] Pires D. Bernard E. M. Pombo J. P. Carmo N. Fialho C. Gutierrez M. G. (2017). Mycobacterium tuberculosis modulates miR-106b-5p to control Cathepsin S expression resulting in higher pathogen survival and poor T-cell activation. Front. Immunol. 8:1819:1819. 10.3389/fimmu.2017.01819 29326705 PMC5741618

[B149] Qi R. Singh D. Kao C. C. (2012). Proteolytic processing regulates Toll-like receptor 3 stability and endosomal localization. J. Biol. Chem. 287 (39), 32617–32629. 10.1074/jbc.M112.387803 22865861 PMC3463343

[B150] Qi X. Man S. M. Malireddi R. K. S. Karki R. Lupfer C. Gurung P. (2016). Cathepsin B modulates lysosomal biogenesis and host defense against *Francisella novicida* infection. J. Exp. Med. 213 (10), 2081–2097. 10.1084/jem.20151938 27551156 PMC5030800

[B151] Rajaram K. Nelson D. E. (2015). *Chlamydia muridarum* infection of macrophages elicits bactericidal nitric oxide production *via* reactive oxygen species and cathepsin B. Infect. Immun. 83 (8), 3164–3175. 10.1128/IAI.00382-15 26015483 PMC4496605

[B152] Reddy V. Y. Zhang Q. Y. Weiss S. J. (1995). Pericellular mobilization of the tissue-destructive cysteine proteinases, cathepsins B, L, and S, by human monocyte-derived macrophages. Proc. Natl. Acad. Sci. U.S.A. 92 (9), 3849–3853. 10.1073/pnas.92.9.3849 7731994 PMC42059

[B153] Reeves E. P. Lu H. Jacobs H. L. Messina C. G. Bolsover S. Gabella G. (2002). Killing activity of neutrophils is mediated through activation of proteases by K+ flux. Nature 416, 291–297. 10.1038/416291a 11907569

[B154] Reich M. Spindler K. D. Burret M. Kalbacher H. Boehm B. O. Burster T. (2010). Cathepsin A is expressed in primary human antigen-presenting cells. Immunol. Lett. 128, 143–147. 10.1016/j.imlet.2009.11.010 19954752

[B155] Ren Y. Khan F. A. Pandupuspitasari N. S. Zhang S. (2017). Immune evasion strategies of pathogens in macrophages: the potential for limiting pathogen transmission. Curr. Issues Mol. Biol. 21, 21–40. 10.21775/cimb.021.021 27033743

[B156] Riese R. J. Wolf P. R. Bromme D. Natkin L. R. Villadangos J. A. Ploegh H. L. (1996). Essential role for cathepsin S in MHC class II-associated invariant chain processing and peptide loading. Immunity 4 (4), 357–366. 10.1016/S1074-7613(00)80249-6 8612130

[B157] Rittig M. R. Alvarez-Martinez M. T. Porte F. Liautard J. P. Rouot B. (2001). Intracellular Survival of *Brucella* spp. in Human Monocytes Involves Conventional Uptake but Special Phagosomes. Infect. Immun. 69 (6), 3995–4006. 10.1128/IAI.69.6.3995-4006.2001 11349069 PMC98462

[B158] Rivera-Marrero C. A. Stewart J. Shafer W. M. Roman J. (2004). The down-regulation of cathepsin G in THP-1 monocytes after infection with *Mycobacterium tuberculosis* is associated with increased intracellular survival of bacilli. Infect. Immun. 72 (10), 5712–5721. 10.1128/IAI.72.10.5712-5721.2004 15385470 PMC517540

[B159] Roberts T. L. Dunn J. A. Terry T. D. Jennings M. P. Hume D. A. Sweet M. J. (2005). Differences in macrophage activation by bacterial DNA and CpG-containing oligonucleotides. J. Immunol. 175 (6), 3569–3576. 10.4049/jimmunol.175.6.3569 16148100

[B160] Rodriguez-Franco E. J. Cantres-Rosario Y. M. Plaud-Valentin M. Romeu R. Rodríguez Y. Skolasky R. (2012). Dysregulation of macrophage-secreted cathepsin B contributes to HIV-1-linked neuronal apoptosis. PloS One 7 (5), e36571. 10.1371/journal.pone.0036571 22693552 PMC3365072

[B161] Rogan M. P. Taggart C. C. Greene C. M. Murphy P. G. O’Neill S. J. McElvaney N. G. (2004). Loss of microbicidal activity and increased formation of biofilm due to decreased lactoferrin activity in patients with cystic fibrosis. J. Infect. Dis. 190 (7), 1245–1253. 10.1086/423821 15346334

[B162] Rossi A. Deveraux Q. Turk B. Sali A. (2004). Comprehensive search for cysteine cathepsins in the human genome. Biol. Chem. 385 (5), 363–372. 10.1515/BC.2004.040 15195995

[B163] Rossman M. D. Maida B. T. Douglas S. D. (1990). Monocyte-derived macrophage and alveolar macrophage fibronectin production and cathepsin D activity. Cell. Immunol. 126 (2), 268–277. 10.1016/0008-8749(90)90320-Q 2107030

[B164] Rovery C. Brouqui P. Raoult D. (2008). Questions on mediterranean spotted fever a century after its discovery. Emerg. Infect. Dis. 14 (9), 1360–1367. 10.3201/eid1409.071133 18760001 PMC2603122

[B165] Russell D. G. Vanderven B. C. Glennie S. Mwandumba H. Heyderman R. S. (2009). The macrophage marches on its phagosome: dynamic assays of phagosome function. Nat. Rev. Immunol. 9, 594–600. 10.1038/nri2591 19590530 PMC2776640

[B166] Sakai H. Saku T. Kato Y. Yamamoto K. (1989). Quantitation and immunohistochemical localization of cathepsins E and D in rat tissues and blood cells. Biochim. Biophys. Acta 991, 367–375. 10.1016/0304-4165(89)90130-x 2655714

[B167] Salcedo S. P. Noursadeghi M. Cohen J. Holden D. W. (2001). Intracellular replication of *Salmonella* typhimurium strains in specific subsets of splenic macrophages *in vivo* . Cell. Microbiol. 3 (9), 587–597. 10.1046/j.1462-5822.2001.00137.x 11553011

[B168] Salpeter S. J. Pozniak Y. Merquiol E. Ben-Nun Y. Geiger T. Blum G. (2015). A novel cysteine cathepsin inhibitor yields macrophage cell death and mammary tumor regression. Oncogene 34, 6066–6078. 10.1038/onc.2015.51 25798843

[B169] Samanta D. Clemente T. M. Schuler B. E. Gilk S. D. (2019). Coxiella burnetii Type 4B Secretion System-dependent manipulation of endolysosomal maturation is required for bacterial growth. PloS Pathog. 15 (12), e1007855. 10.1371/journal.ppat.1007855 31869379 PMC6953889

[B170] Sanman L. E. van der Linden W. A. Verdoes M. Bogyo M. (2016). Bifunctional probes of cathepsin protease activity and pH reveal alterations in endolysosomal pH during bacterial infection. Cell Chem. Biol. 23 (7), 793–804. 10.1016/j.chembiol.2016.05.019 27427229 PMC4982764

[B171] Santic M. Akimana C. Asare R. Kouokam J. C. Atay S. Kwaik Y. A. (2009). Intracellular fate of *Francisella tularensis* within arthropod-derived cells. Environ. Microbiol. 11 (6), 1473–1481. 10.1111/j.1462-2920.2009.01875.x 19220402

[B172] Santic M. Al-Khodor S. Abu Kwaik Y. (2010). Cell biology and molecular ecology of *Francisella tularensis* . Cell. Microbiol. 12 (2), 129–139. 10.1111/j.1462-5822.2009.01400.x 19863554

[B173] Sarkar A. Tindle C. Pranadinata R. F. Reed S. Eckmann L. Stappenbeck T. S. (2017). ELMO1 regulates autophagy induction and bacterial clearance during enteric infection. J. Infect. Dis. 216 (12), 1655–1666. 10.1093/infdis/jix528 29029244 PMC5853658

[B174] Sastradipura D. F. Nakanishi H. Tsukuba T. Nishishita K. Sakai H. Kato Y. (1998). Identification of cellular compartments involved in processing of cathepsin E in primary cultures of rat microglia. J. Neurochem. 70, 2045–2056. 10.1046/j.1471-4159.1998.70052045.x 9572291

[B175] Satake A. Itoh K. Shimmoto M. Saido T. C. Sakuraba H. Suzuki Y. (1994). Distribution of lysosomal protective protein in human tissues. Biochem. Biophys. Res. Commun. 205 (1), 38–43. 10.1006/bbrc.1994.2626 7999052

[B176] Schimmelpfeng L. Langenberg U. Hinrich Peters J. (1980). Macrophages overcome mycoplasma infections of cells *in vitro* . Nature 285 (5767), 661–662. 10.1038/285661a0 7393319

[B177] Schroeder G. N. Hilbi H. (2007). Cholesterol is required to trigger caspase-1 activation and macrophage apoptosis after phagosomal escape of Shigella. Cell. Microbiol. 9 (1), 265–278. 10.1111/j.1462-5822.2006.00787.x 16925787

[B178] Sealy L. Mota F. Rayment N. Tatnell P. Kay J. Chain B. (1996). Regulation of cathepsin E expression during human b cell differentiation *in vitro* . Eur. J. Immunol. 26 (8), 1838–1843. 10.1002/eji.1830260826 8765029

[B179] Sedor J. Hogue L. Akers K. Boslaugh S. Schreiber J. Ferkol T. (2007). Cathepsin-G interferes with clearance of *Pseudomonas aeruginosa* from mouse lungs. Pediatr. Res. 61 (1), 26–31. 10.1203/01.pdr.0000250043.90468.c2 17211136

[B180] Selkrig J. Li N. Hausmann A. Mangan M. S. J. Zietek M. Mateus A. (2020). Spatiotemporal proteomics uncovers cathepsin-dependent macrophage cell death during *Salmonella* infection. Nat. Microbiol. 5, 1119–1133. 10.1038/s41564-020-0736-7 32514074 PMC7610801

[B181] Sendide K. Deghmane A. E. Reyrat J. M. Talal A. Hmama Z. (2004). *Mycobacterium bovis* BCG urease attenuates major histocompatibility complex class II trafficking to the macrophage cell surface. Infect. Immun. 72 (7), 4200–4209. 10.1128/IAI.72.7.4200-4209.2004 15213164 PMC427455

[B182] Sendide K. Deghmane A. E. Pechkovsky D. Av-Gay Y. Talal A. Hmama Z. (2005). *Mycobacterium bovis* BCG attenuates surface expression of mature class II molecules through IL-10-dependent inhibition of cathepsin S. J. Immunol. 175 (8), 5324–5332. 10.4049/jimmunol.175.8.5324 16210638

[B183] Shafer W. M. Onunka V. C. Martin L. E. (1986). Antigonococcal activity of human neutrophil cathepsin G. Infect. Immun. 54 (1), 184–188. 10.1128/IAI.54.1.184-188.1986 3093384 PMC260134

[B184] Shafer W. M. Onunka V. C. Jannoun M. Huthwaite L. W. (1990). Molecular mechanism for the antigonococcal action of lysosomal cathepsin G. Mol. Microbiol. 4 (8), 1269–1277. 10.1111/j.1365-2958.1990.tb00706.x 2126324

[B185] Shafer W. M. Morse S. A. (1987). Cleavage of the protein III and major iron-regulated protein of *Neisseria gonorrhoeae* by lysosomal cathepsin G. J. Gen. Microbiol. 133 (1), 155–162. 10.1099/00221287-133-1-155 3116158

[B186] Sharma T. Grover S. Arora N. Manjunath P. Ehtesham N. Z. Hasnain S. E. (2020). PGRS domain of Rv0297 of Mycobacterium tuberculosis is involved in modulation of macrophage functions to favor bacterial persistence. Front. Cell. Infect. Microbiol. 10, 451. 10.3389/fcimb.2020.00451 33042856 PMC7517703

[B187] Shi G. P. Chapman H. A. Bhairi S. M. DeLeeuw C. Reddy V. Y. Weiss S. J. (1995). Molecular cloning of human cathepsin o, a novel endoproteinase and homologue of rabbit OC2. FEBS Lett. 357 (2), 129–134. 10.1016/0014-5793(94)01349-6 7805878

[B188] Shi G. P. Bryant R. A. R. Riese R. Verhelst S. Driessen C. Li Z. (2000). Role for cathepsin F in invariant chain processing and major histocompatibility complex class II peptide loading by macrophages. J. Exp. Med. 191 (7), 1177–1186. 10.1084/jem.191.7.1177 10748235 PMC2193169

[B189] Shlomo S. B. Mouhadeb O. Cohen K. Varol C. Gluck N. (2019). COMMD10-guided phagolysosomal maturation promotes clearance of *Staphylococcus aureus* in macrophages. iScience 14, 147–163. 10.1016/j.isci.2019.03.024 30959277 PMC6453835

[B190] Singh C. R. Moulton R. A. Armitige L. Y. Bidani A. Snuggs M. Dhandayuthapani S. (2006). Processing and presentation of a mycobacterial antigen 85B epitope by murine macrophages is dependent on the phagosomal acquisition of vacuolar proton ATPase and *in situ* activation of cathepsin D. J. Immunol. 177 (5), 3250–3259. 10.4049/jimmunol.177.5.3250 16920965

[B191] Sintiprungrat K. Singhto N. Sinchaikul S. Chen S. T. Thongboonkerd V. (2010). Alterations in cellular proteome and secretome upon differentiation from monocyte to macrophage by treatment with phorbol myristate acetate: insights into biological processes. J. Proteomics 73 (3), 602–618. 10.1016/j.jprot.2009.08.001 19683082

[B192] Skvarc M. Stubljar D. Kopitar A. N. Jeverica S. Tepes B. Kos J. (2013). Inhibition of cathepsin X enzyme influences the immune response of THP-1 cells and dendritic cells infected with Helicobacter pylori. Radiol. Oncol. 47 (3), 258–265. 10.2478/raon-2013-0043 24133391 PMC3794882

[B193] Slauch J. M. (2011). How does the oxidative burst of macrophages kill bacteria? Still an open question. Mol. Microbiol. 80 (3), 580–583. 10.1111/j.1365-2958.2011.07612.x 21375590 PMC3109634

[B194] Snowden J. Bhimji S. S. (2018). Rickettsial infection (Treasure Island, FL: Stat Pearls).

[B195] Soualhine H. Deghmane A. E. Sun J. Mak K. Talal A. Av-Gay Y. (2007). *Mycobacterium bovis* bacillus Calmette-Guerin secreting active cathepsin S stimulates expression of mature MHC class II molecules and antigen presentation in human macrophages. J. Immunol. 179 (8), 5137–5145. 10.4049/jimmunol.179.8.5137 17911599

[B196] Srivastava M. Meinders A. Steinwede K. Maus R. Lucke N. Bühling F. (2006). Mediator responses of alveolar macrophages and kinetics of mononuclear phagocyte subset recruitment during acute primary and secondary mycobacterial infections in the lungs of mice. Cell. Microbiol. 9 (3), 738–752. 10.1111/j.1462-5822.2006.00824.x 17054437

[B197] Standish A. J. Weiser J. N. (2009). Human neutrophils kill *Streptococcus pneumoniae via* serine proteases. J. Immunol. 183 (4), 2602–2609. 10.4049/jimmunol.0900688 19620298

[B198] Starr T. Ng T. W. Wehrly T. D. Knodler L. A. Celli J. (2008). Brucella intracellular replication requires trafficking through the late endosomal/lysosomal compartment. Traffic 9 (5), 678–694. 10.1111/j.1600-0854.2008.00718.x 18266913

[B199] Steele-Mortimer O. (2008). The *Salmonella*-containing vacuole – moving with the times. Curr. Opin. Microbiol. 11 (1), 38–45. 10.1016/j.mib.2008.01.002 18304858 PMC2577838

[B200] Steinwede K. Maus R. Bohling J. Voedisch S. Braun A. Ochs M. (2012). Cathepsin G and neutrophil elastase contribute to lung-protective immunity against mycobacterial infections in mice. J. Immunol. 188 (9), 4476–4487. 10.4049/jimmunol.1103346 22461690

[B201] Sukhova G. K. Zhang Y. Pan J. H. Wada Y. Yamamoto T. Naito M. (2003). Deficiency of cathepsin S reduces atherosclerosis in LDL receptor–deficient mice. J. Clin. Invest. 111 (6), 897–906. 10.1172/JCI14915 12639996 PMC153760

[B202] Tan B. H. Meinken C. Bastian M. Bruns H. Legaspi A. Ochoa M. T. (2006). Macrophages acquire neutrophil granules for antimicrobial activity against intracellular pathogens. J. Immunol. 177 (3), 1864–1871. 10.4049/jimmunol.177.3.1864 16849498

[B203] Tianqian Z. Yoichi M. Tohru S. Yoko N. Kazunari I. Hajime H. (2001). Treatment with cathepsin L inhibitor potentiates Th2-type immune response in *Leishmania major*—infected BALB/c mice. Int. Immunol. 13 (8), 975–982. 10.1093/intimm/13.8.975 11470767

[B204] Tietzel I. Quayle A. J. Carabeo R. A. (2019). Alternatively activated macrophages are host cells for *Chlamydia trachomatis* and reverse anti-chlamydial classically activated macrophages. Front. Microbiol. 10:919:919. 10.3389/fmicb.2019.00919 31134002 PMC6524708

[B205] Tomberg J. Fedarovich A. Vincent L. R. Jerse A. E. Unemo M. Davies C. (2017). Alanine-501 mutations in penicillin-binding protein 2 from *Neisseria gonorrhoeae*: structure, mechanism, and effects on cephalosporin resistance and biological fitness. Biochemistry 56 (8), 1140–1150. 10.1021/acs.biochem.6b01030 28145684 PMC5502787

[B206] Toscano F. Estornes Y. Virard F. Garcia-Cattaneo A. Pierrot A. Vanbervliet B. (2013). Cleaved/associated TLR3 represents the primary form of the signaling receptor. J. Immunol. 190 (2), 764–773. 10.4049/jimmunol.1202173 23255358

[B207] Tranchemontagne Z. R. Camire R. B. O’Donnell V. J. Baugh J. Burkholder K. M. (2016). *Staphylococcus aureus* strain USA300 perturbs acquisition of lysosomal enzymes and requires phagosomal acidification for survival inside macrophages. Infect. Immun. 84 (1), 241–253. 10.1128/IAI.00704-15 26502911 PMC4694005

[B208] Tripp C. S. Wolf S. F. Unanue E. R. (1993). Interleukin 12 and tumor necrosis factor alpha are costimulators of interferon gamma production by natural killer cells in severe combined immunodeficiency mice with listeriosis, and interleukin 10 is a physiologic antagonist. Proc. Natl. Acad. Sci. U.S.A. 90 (8), 3725–3729. 10.1073/pnas.90.8.3725 8097322 PMC46374

[B209] Tsukuba T. Yamamoto S. Yanagawa M. Okamoto K. Okamoto Y. Nakayama K. II (2006). Cathepsin E-deficient mice show increased susceptibility to bacterial infection associated with the decreased expression of multiple cell surface Toll-like receptors. J. Biochem. 140 (1), 57–66. 10.1093/jb/mvj132 16877769

[B210] Tsukuba T. Yanagawa M. Kadowaki T. Takii R. Okamoto Y. Sakai E. (2013). Cathepsin E deficiency impairs autophagic proteolysis in macrophages. PloS One 8 (12), e82415. 10.1371/journal.pone.0082415 24340026 PMC3855462

[B211] Turk V. Turk B. Turk D. (2001). Lysosomal cysteine proteases: facts and opportunities. EMBO J. 20 (17), 4629–4633. 10.1093/emboj/20.17.4629 11532926 PMC125585

[B212] Turk B. Turk D. Salvesen G. S. (2002). Regulating cysteine protease activity: essential role of protease inhibitors as guardians and regulators. Curr. Pharm. Des. 8, 1623–1637. 10.2174/1381612023394124 12132995

[B213] Turk V. Stoka V. Vasiljeva O. Renko M. Sun T. Turk B. (2012). Cysteine cathepsins: from structure, function and regulation to new frontiers. Biochim. Biophys. Acta 1824 (1), 68–88. 10.1016/j.bbapap.2011.10.002 22024571 PMC7105208

[B214] Uchiyama Y. (2001). Autophagic cell death and its execution by lysosomal cathepsins. Arch. Histol. Cytol. 64 (3), 233–246. 10.1679/aohc.64.233 11575420

[B215] Unanue E. R. (1997). Inter-relationship among macrophages, natural killer cells and neutrophils in early stages of Listeria resistance. Curr. Op. Immunol. 9 (1), 35–43. 10.1016/s0952-7915(97)80156-2 9039774

[B216] Vazquez-Torres A. Jones-Carson J. Bäumler A. J. Falkow S. Valdivia R. Brown W. (1999). Extraintestinal dissemination of Salmonella by CD18-expressing phagocytes. Nature 401 (6755), 804–808. 10.1038/44593 10548107

[B217] Villani A. C. Satija R. Reynolds G. Sarkizova S. Shekhar K. Fletcher J. (2017). Single-cell RNA-seq reveals new types of human blood dendritic cells, monocytes and progenitors. Science 356 (6335), eaah4573. 10.1126/science.aah4573 28428369 PMC5775029

[B218] Voth D. E. Heinzen R. A. (2007). Lounging in a lysosome: the intracellular lifestyle of. Coxiella Burnetii. Cell. Microbiol. 9 (4), 829–840. 10.1111/j.1462-5822.2007.00901.x 17381428

[B219] Vural A. Kehrl J. H. (2014). Autophagy in macrophages: impacting inflammation and bacterial infection. Scientifica 2014:825463. 10.1155/2014/825463 24818040 PMC4000662

[B220] Wang Z. Sun D. Chen G. Li G. Dou S. Wang R. (2017). Tim-3 inhibits macrophage control of *Listeria monocytogenes* by inhibiting Nrf2. Sci. Rep. 7:42095. 10.1038/srep42095 28205579 PMC5311873

[B221] Welin A. Eklund D. Stendahl O. Lerm M. (2011). Human macrophages infected with a high burden of ESAT-6-expressing M. tuberculosis undergo caspase-1- and cathepsin B-independent necrosis. PloS One 6 (5), e20302. 10.1371/journal.pone.0020302 21637850 PMC3102687

[B222] Wex T. Buhling F. Wex H. Gunther D. Malfertheiner P. Weber E. (2001). Human Cathepsin W, a cysteine protease predominantly expressed in NK cells, is mainly localized in the endoplasmic reticulum. J. Immunol. 167, 2172–2178. 10.4049/jimmunol.167.4.2172 11490002

[B223] Willstätter R. Bamann E. (1929). Über die Proteasen der Magenschleimhaut. Erste Abhandlung über die Enzyme der Leukocyten. Hoppe-Seyler’s Z. Physiol. Chem. 180, 127–143. 10.1515/bchm2.1929.180.1-3.127

[B224] Woischnik M. Bauer A. Aboutaam R. Pamir A. Stanzel F. de Blic J. (2008). Cathepsin H and napsin A are active in the alveoli and increased in alveolar proteinosis. Eur. Respir. J. 31 (6), 1197–1204. 10.1183/09031936.00081207 18216060

[B225] Xavier M. N. Winter M. G. Spees A. M. Nguyen K. Atluri V. L. Silva T. M. A. (2013). CD4+ T cell-derived IL-10 promotes *Brucella abortus* persistence *via* modulation of macrophage function. PloS Pathog. 9 (6), e1003454. 10.1371/journal.ppat.1003454 23818855 PMC3688575

[B226] Xia H. H. X. Lam S. K. Huang X. R. Wong W. M. Leung S. Y. Yuen S. T. (2004). *Helicobacter pylori* infection is associated with increased expression of macrophage migration inhibitory factor by epithelial cells, T-cells and macrophages in gastric mucosa. J. Infect. Dis. 190 (2), 293–302. 10.1086/421915 15216464

[B227] Xu X. Greenland J. Baluk P. Adams A. Bose O. McDonald D. M. (2013). Cathepsin L protects mice from mycoplasmal infection and is essential for airway lymphangiogenesis. Am. J. Respir. Cell. Mol. Biol. 49 (3), 437–444. 10.1165/rcmb.2013-0016OC 23600672 PMC3824055

[B228] Yadati T. Houben T. Bitorina A. Shiri-Sverdlov R. (2020). The ins and outs of cathepsins: Physiological function and role in disease management. Cells 9 (7):1679. 10.3390/cells9071679 PMC7407943 32668602

[B229] Yasuda Y. Li Z. Greenbaum D. Bogyo M. Weber E. Bromme D. (2004). Cathepsin V, a novel and potent elastolytic activity expressed in activated macrophages. J. Biol. Chem. 279, 36761– 36770. 10.1074/jbc.M403986200 15192101

[B230] Zavasnik-Bergant T. Turk B. (2007). Cysteine proteases: destruction ability versus immunomodulation capacity in immune cells. Biol. Chem. 388 (11), 1141–1149. 10.1515/BC.2007.144 17976006

[B231] Zhang T. Maekawa Y. Hanba J. Dainichi T. Nashed B. F. Hisaeda H. (2000). Lysosomal cathepsin B plays an important role in antigen processing, while cathepsin D is involved in degradation of the invariant chain in ovalbumin-immunized mice. Immunology 100 (1), 13–20. 10.1046/j.1365-2567.2000.00000.x 10809954 PMC2326990

[B232] Zhang N. Gao P. Yin B. Li J. Wu T. Kuang Y. (2019). Cathepsin L promotes secretory IgA response by participating in antigen presentation pathways during *Mycoplasma Hyopneumoniae* infection. PloS One 14 (4), e0215408. 10.1371/journal.pone.0215408 30986254 PMC6464228

